# A Hybrid MCDM and Machine Learning Framework for Thalassemia Risk Assessment in Pregnant Women

**DOI:** 10.3390/diagnostics15222833

**Published:** 2025-11-08

**Authors:** Shefayatuj Johara Chowdhury, Tanjim Mahmud, Farzana Tasnim, Sanjida Sharmin, Saida Nawal, Umme Habiba Papri, Samia Afreen Dolon, Md. Eftekhar Alam, Mohammad Shahadat Hossain, Karl Andersson

**Affiliations:** 1 Department of Computer Science and Engineering, International Islamic University Chittagong, Chittagong 4318, Bangladesh; shefaya61@gmail.com (S.J.C.); farzanatasnim34@gmail.com (F.T.); ssharmin114@gmail.com (S.S.); nawal.parisha017@gmail.com (S.N.); ummeha65@gmail.com (U.H.P.); samiaafreendolon@gmail.com (S.A.D.); 2 Department of Computer Science and Engineering, Rangamati Science and Technology University, Rangamati 4500, Bangladesh; 3 Department of Electrical and Electronic Engineering, International Islamic University Chittagong, Chittagong 4318, Bangladesh; eftekhar78@gmail.com; 4 Department of Computer Science and Engineering, University of Chittagong, Chittagong 4331, Bangladesh; hossain_ms@cu.ac.bd; 5 Cybersecurity Laboratory, Luleå University of Technology, S-931 87 Skellefteå, Sweden; karl.andersson@ltu.se

**Keywords:** thalassemia prediction, machine learning, explainable AI, SHAP, LIME, AHP-TOPSIS, feature selection, clinical decision support, CatBoost, maternal health, biomedical informatics, risk assessment

## Abstract

**Background:** Thalassemia has been recognized as a critical public health issue in Bangladesh, especially among pregnant women, due to its hereditary nature and the lack of early screening infrastructure. Early identification of at-risk individuals is essential to prevent the transmission of this genetic disorder to future generations and to reduce the burden on an already strained healthcare system. **Methods:** In this study, an innovative framework for thalassemia risk assessment has been developed by integrating Multi-Criteria Decision-Making (MCDM) methods—specifically AHP-TOPSIS—with machine learning algorithms including Random Forest, XGBoost, and CatBoost. Explainable Artificial Intelligence (XAI) techniques such as SHAP and LIME have also been incorporated to improve model transparency and trustworthiness. Real-world clinical and demographic data, consisting of 16 features and 1200 samples, have been collected through a structured survey and processed using rigorous feature selection and ranking methods. Risk stratification has been performed to classify patients into high, medium, and low categories, enabling targeted intervention. **Results:** Among all models, the XGBoost classifier trained on AHP–TOPSIS–prioritized features achieved a consistent accuracy of 99.28% under stratified 20-fold cross-validation, demonstrating robust diagnostic classification performance. The model predominantly captures hematologic patterns characteristic of thalassemia manifestations, functioning as an assistive diagnostic framework rather than a causal risk predictor. The explainability of predictions, ensured through comprehensive visual and statistical analyses, further enhances the model’s clinical transparency and reliability. **Conclusions:** The proposed MCDM–machine learning framework demonstrates strong potential for improving thalassemia risk assessment, enabling early detection and informed decision-making in maternal healthcare. The proposed framework should be regarded as a preliminary proof-of-concept system that demonstrates the feasibility of integrating Multi-Criteria Decision-Making (AHP–TOPSIS) with advanced machine learning and explainable-AI techniques for thalassemia assessment. Although the model achieved strong diagnostic performance under nested cross-validation, additional external validation and inclusion of causal predictors are required before clinical deployment.

## 1. Introduction

Thalassemia is not just a medical condition; it represents a lifelong challenge for countless families in Bangladesh. This inherited blood disorder impairs the body’s ability to produce hemoglobin, leading to chronic anemia, profound fatigue, and a range of severe health complications [1]. The two main types of thalassemia, α-thalassemia and β-thalassemia, with β-thalassemia being more widespread and severe, contribute to the high burden of this disorder in the country [2]. While thalassemia is common in regions like Southeast Asia, the Mediterranean, and India, Bangladesh faces distinct challenges due to limited awareness, inadequate healthcare infrastructure, and the absence of regular screening programs [3].

For pregnant women, the risks associated with thalassemia are particularly concerning [4]. Many expectant mothers are unaware that they carry the thalassemia gene, unknowingly passing it to their children. This lack of awareness can lead to devastating outcomes, including miscarriage, stillbirth, low birth weight, and severe anemia in newborns [5]. In rural areas, where healthcare access is already limited, the absence of early diagnosis and treatment compounds these challenges, leaving many families struggling with preventable complications. Without timely intervention, thalassemia significantly impacts maternal and child health, putting further strain on an already overburdened healthcare system [6].

Addressing this issue requires a transformative approach. This study proposes an innovative framework that integrates multi-criteria decision-making (MCDM) techniques [7,8], such as AHP-TOPSIS [9], with machine learning models to improve diagnostic accuracy and risk assessment. The incorporation of Explainable Artificial Intelligence (XAI) techniques [10], such as SHAP and LIME, enhances transparency, enabling healthcare professionals to better understand and trust the predictions of the model. By utilizing real-world clinical and demographic data, this research aims to develop a practical, scalable solution tailored to the specific needs of Bangladesh’s healthcare system.

By bridging the gap between technology and healthcare, this study aspires to provide a reliable and cost-effective solution for improving thalassemia screening, especially for pregnant women who are most at risk. Early diagnosis and accurate risk assessment can save lives, prevent complications, and significantly enhance maternal and child health outcomes across the nation.

### 1.1. Research Questions

This study aims to address the existing gap in thalassemia diagnosis and risk assessment by focusing on improving the screening process for pregnant women in Bangladesh. The following research questions will guide the investigation:

- RQ1: What are the key factors affecting the diagnostic accuracy and risk assessment of thalassemia in pregnant women in Bangladesh?
- RQ2: How can a combination of multi-criteria decision-making (MCDM) techniques (AHP-TOPSIS) and machine learning models enhance thalassemia screening in Bangladesh’s healthcare system?
- RQ3: In what ways can the integration of Explainable Artificial Intelligence (XAI) methods like SHAP and LIME improve the interpretability and trustworthiness of thalassemia predictions for healthcare providers?

### 1.2. Motivations

The primary motivation for this research arises from the pressing need to address the challenges of thalassemia screening in Bangladesh, particularly for pregnant women who are most vulnerable. Despite the importance of early diagnosis, current practices are hindered by limited awareness and a lack of technological integration in healthcare systems. The aim of this study is to bridge these gaps by providing a data-driven, machine learning-enhanced framework for thalassemia risk assessment.

### 1.3. Novelty and Contributions

The present study has been designed to advance existing AHP–TOPSIS–ML frameworks through the following contributions:

1. All preprocessing, SelectKBest filtering, model-based importance (mean |SHAP|), AHP weighting, and TOPSIS ranking have been executed within the training folds of a repeated stratified or nested cross-validation, and performance has been evaluated exclusively on the held-out folds.
2. Causal or antecedent predictors (e.g., family history, socioeconomic context) have been distinguished from concurrent hematologic manifestations (e.g., MCV, RDW, Hb), and separate Etiology and Diagnostic models have been assessed to prevent label leakage and clarify their intended applications.
3. Feature-influence differences have been validated beyond SHAP and LIME visualizations by applying the Kruskal–Wallis and pairwise Mann–Whitney U tests, and statistical significance has been confirmed through a SHAP *p*-value heatmap.
4. A Combined Importance Index (normalized SHAP ⊕ SelectKBest) has been formulated and integrated with AHP (ensuring CR<0.1) and TOPSIS so that final feature weights have been derived in an auditable and transparent manner.

The paper is organized as follows: Section 2 provides a comprehensive related works, discussing the current state of research on thalassemia screening, diagnostic techniques, and the application of machine learning and decision-making frameworks in healthcare, with a particular focus on maternal health. Section 3 outlines the methodology adopted in this study, including details on data collection, the integration of AHP-TOPSIS for ranking critical success factors, the application of machine learning models for predictive analysis, and the use of Explainable Artificial Intelligence (XAI) techniques such as SHAP and LIME to improve model interpretability. In Section 4, the experimental results are presented, including the evaluation of the proposed framework’s performance, accuracy, and the impact of XAI methods on trustworthiness and decision-making. Finally, Section 5 concludes the paper by summarizing the key findings, discussing their practical implications for improving thalassemia screening in Bangladesh, and suggesting possible directions for future research.

## 2. Related Works

Thalassemia remains a pressing public health concern, especially in regions with a high prevalence of inherited genetic disorders. A wide range of studies has focused on improving early diagnosis [11], risk prediction [8], and disease management strategies [12]. Central to these efforts is the integration of clinical, genetic, and demographic data to strengthen risk assessment models—particularly for vulnerable populations such as pregnant women. In parallel, significant attention has been given to the development of predictive models that address critical challenges, including data imbalance, feature selection, and the need for hybrid analytical approaches.

Recent advancements in machine learning (ML) and multi-criteria decision-making (MCDM) techniques have significantly enhanced diagnostic accuracy and decision support systems in medical informatics. Researchers have increasingly integrated ML algorithms with decision-analysis frameworks to improve the interpretability, scalability, and cost-effectiveness of healthcare solutions across a variety of clinical domains.

Machine learning has also been applied to study anemia in pregnant women. In Ethiopia, a predictive model was developed to assess the prevalence of anemia, identifying rural residency and lower education levels as significant risk factors. The use of decision trees and random forests improved prediction accuracy, highlighting the importance of targeted interventions to enhance maternal healthcare and support evidence-based management strategies for anemia prevention [13]. Table 1 presents a comparative summary of recent studies in disease prediction and screening, highlighting their domains, methodologies, datasets, performance metrics, and identified research gaps. Overall, existing research continues to face challenges related to achieving high accuracy, generalizability, and resource efficiency in medical AI applications. This study builds on previous work by proposing a comprehensive methodology tailored to the unique healthcare and socioeconomic landscape of Bangladesh. Through the integration of machine learning, AHP-TOPSIS, and Explainable AI (XAI), this research aims to bridge critical gaps in thalassemia risk prediction and management, contributing to more effective and accessible screening solutions [14,15]. The current study outlines significant advancements in thalassemia risk assessment and prenatal care, particularly in resource-constrained settings. In study  [14] proposed a supervised machine learning model to identify β-thalassemia carriers using complete blood count (CBC) data. By applying oversampling techniques such as SMOTE and ADASYN to address class imbalance, the authors achieved a promising accuracy of 96%. Feature reduction through PCA and SVD further improved performance, contributing to early-stage diagnosis.

Dejene et al. [13] applied ensemble ML techniques to predict anemia levels among pregnant women in Ethiopia. Using demographic data from over 11,000 participants, the CatBoost algorithm yielded the highest accuracy (97.6%). The study emphasized socioeconomic factors like education and residence, demonstrating the significance of integrating contextual features into predictive modeling.

In the context of mass screening for β-thalassemia traits, Jain et al. [16] evaluated the effectiveness of 42 RBC-based formulas using multi-criteria decision-making (MCDM) approaches. The newly developed SCS BTT formula, validated using TOPSIS and COPRAS, showed 100% sensitivity when the mean corpuscular volume (MCV) was below 80 fL. The authors advocate its use in low-resource settings due to affordability and accuracy.

The CWBCM method, introduced by Parishani and Rasti-Barzoki [17], enhances classifier selection by integrating confusion matrix metrics (accuracy, sensitivity, and specificity) within MCDM frameworks like AHP and Shannon Entropy. Applied to datasets on COVID-19, diabetes, and thyroid disease, CWBCM demonstrated improved decision quality compared to traditional weighting methods.

For breast cancer detection, Mustapha et al. [18] combined AHP and TOPSIS with supervised learning models using the Wisconsin Diagnostic Breast Cancer dataset. Their method incorporated not only prediction performance but also deployment feasibility, offering a more holistic evaluation framework for model selection.

Stenwig et al. [19] addressed interpretability concerns in predictive healthcare models. Using SHAP values, they analyzed model behavior across random forests, logistic regression, naive Bayes, and AdaBoost on the eICU dataset. Although the models had similar predictive capacities, the study revealed disparities in how each algorithm processed input features, underlining the importance of explainable AI in clinical practice.

## 3. Methods

The goal of this study is to build an intelligent and transparent decision-support framework for thalassemia risk assessment by integrating AHP, TOPSIS, machine learning, and explainable AI. AHP is used to assign weights to key risk factors based on expert judgment, while TOPSIS ranks these factors to identify high-risk individuals. The ranked dataset is then used to train various machine learning models, improving prediction accuracy and consistency. To ensure interpretability and trust in the results, explainable AI techniques like SHAP and LIME are applied, offering clear insights into how each model arrives at its predictions. This integrated approach provides a reliable, data-driven solution tailored for clinical decision-making.

### 3.1. Workflow Overview

**Figure 1:**
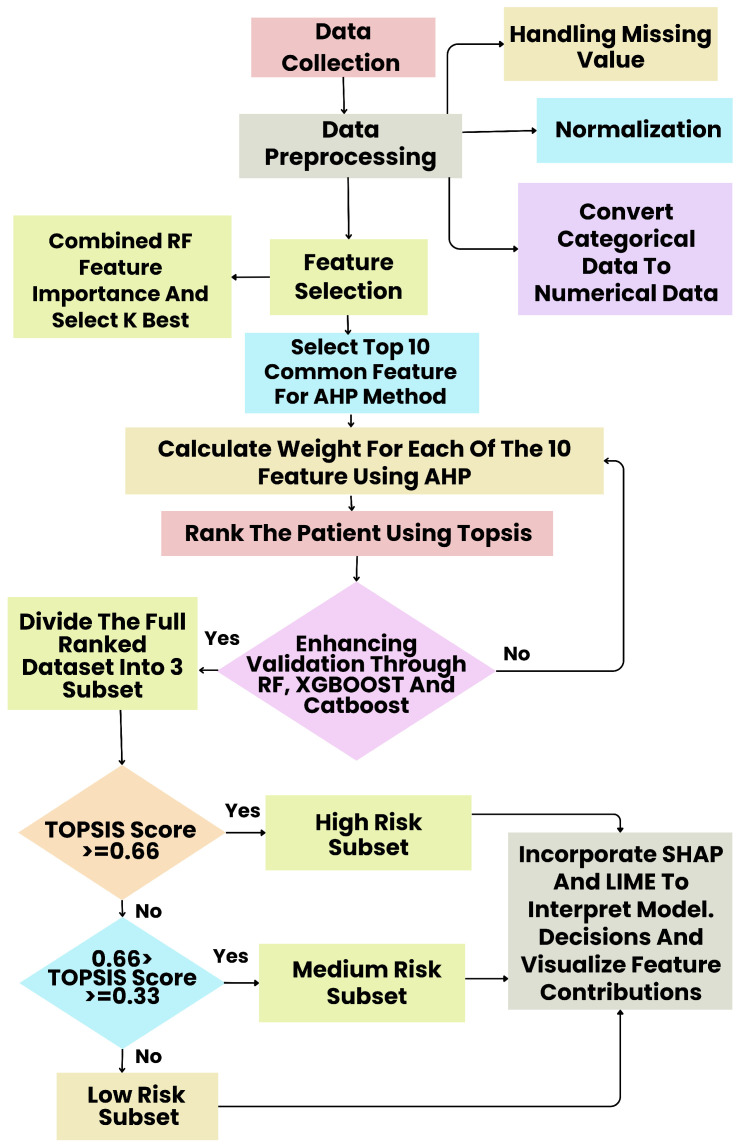
Proposed Method.

The proposed framework establishes a comprehensive and intelligent pipeline for thalassemia diagnostic classification by integrating conventional machine learning techniques, multi-criteria decision-making (AHP–TOPSIS), and explainable AI (XAI). The overall process, illustrated in Figure 1, has been structured into several sequential phases as follows.

Step 1: Data Collection

Clinical and hematological data, including complete blood count (CBC) indices and demographic features, were collected from verified medical centers to ensure diagnostic reliability and diversity of samples.

Step 2: Data Preprocessing

Preprocessing ensured data quality and suitability for machine learning. Missing values were handled through appropriate imputation, numerical features were normalized, and categorical attributes were encoded into numerical form.

Step 3: Feature Selection

A hybrid feature-selection strategy was employed using Random Forest [20] and SelectKBest [21]. The importance scores from both techniques were min–max normalized and averaged to produce a unified ranking. The top ten features were selected for further prioritization through the Analytic Hierarchy Process (AHP).

Step 4: AHP–TOPSIS Based Prioritization

To integrate expert judgment, the AHP method [22] was used to derive feature weights, which were subsequently applied in the TOPSIS framework to compute ranked risk scores for each patient. This combination provided both data-driven and expert-informed weighting for model input preparation.

Step 5: Risk Stratification and Model Training

Based on TOPSIS scores, patients were stratified into three diagnostic categories: High Risk (≥0.66), Medium Risk (0.33–0.66), and Low Risk (<0.33). Machine learning models, including Random Forest, XGBoost, and CatBoost, were trained on the ranked dataset. To ensure methodological rigor, a stratified 20-fold cross-validation scheme was adopted, with feature selection and AHP–TOPSIS weighting performed within each training fold and evaluation restricted to the held-out fold.

Step 6: Explainable AI Integration

Interpretability was achieved using SHAP and LIME, which provided global and local explanations of model behavior, respectively. These visual and statistical interpretations enabled clinicians to understand the influence of hematologic parameters on diagnostic outcomes, enhancing transparency and clinical trust.

### 3.2. Survey Design for Data Collection

To facilitate the early identification and assessment of thalassemia risk, we developed a comprehensive, structured survey that captures a broad range of demographic, clinical, genetic, and socioeconomic information. The questionnaire was designed with guidance from specialists in hematology, genetics, and public health to ensure that all relevant diagnostic indicators are covered. The survey has consisted of sixteen carefully selected features with potential associations to thalassemia diagnosis or carrier status. These include age, Body Mass Index (BMI), hemoglobin level, and major hematologic indices such as Hematocrit (Hct), Mean Corpuscular Volume (MCV), Mean Corpuscular Hemoglobin (MCH), Mean Corpuscular Hemoglobin Concentration (MCHC), Red Cell Distribution Width (RDW), and RBC count. In addition, hereditary risk factors such as family history of thalassemia and the presence of genetic markers were included (see Table 2).

Recognizing the influence of social and environmental factors on health outcomes, the questionnaire has also collected information on socioeconomic status, education level, and residential location (urban or rural). Parity—the number of times a woman has given birth—has been included as a reproductive health indicator. Each question has been clearly structured using a mix of multiple-choice, yes/no, and percentage-based response formats. For instance, participants have been asked to estimate the prevalence of thalassemia within their families using a percentage input. The survey was pilot-tested on a small representative sample to refine question clarity and ensure usability before large-scale deployment. In total, responses from 1200 pregnant women have been collected for analysis.

All participants were informed about the study objectives and voluntarily provided consent prior to completing the questionnaire. No personally identifying information was recorded, and all data were anonymized to maintain confidentiality. Although data were gathered from several diagnostic centers to ensure clinical and population diversity, regional bias may remain since most respondents were from the Chattogram Division. This limitation has been acknowledged in the discussion section. Nevertheless, the structured survey provides a reliable and representative foundation for machine learning–based diagnostic modeling of thalassemia risk (see Table 3).

### 3.3. Feature Engineering

Feature engineering plays a crucial role in enhancing the efficiency and accuracy of machine learning models by eliminating irrelevant or redundant attributes. In this study, two well-established techniques—Random Forest Feature Importance and SelectKBest—were employed to identify the most significant features contributing to the prediction of thalassemia. These methods were chosen to provide both a model-based and a statistical perspective on feature relevance. While Random Forest evaluates the contribution of each feature based on its impact on predictive performance, SelectKBest ranks features using statistical tests that measure class separability. The integration of both methods ensured a robust and comprehensive feature selection process.

#### 3.3.1. Random Forest Feature Importance Method

Random Forest, an ensemble learning algorithm composed of multiple decision trees, was utilized to estimate the importance of each feature. During the training phase, the algorithm constructs numerous trees, where each node is split based on a feature that minimizes an impurity criterion. The decrease in impurity—measured either by Gini impurity or entropy—was aggregated to determine the contribution of each feature.

The impurity measures are defined as follows:

(1) $$\mathrm{Gini}\;\mathrm{Impurity}:\;Gini=1-\sum_{k}P_{k}^{2}$$

(2) $$\mathrm{Entropy}:\;Entropy=-\sum_{k}p_{k}\log_{2}p_{k}$$

where $P_{k}$ represents the proportion of samples belonging to class *k*.

The importance score for a feature *j* was computed by summing the impurity reductions attributed to that feature across all trees in the forest:

(3) $$FI_{j}=\sum_{t=1}^{T}I_{tj}\cdot {\mathrm{Impurity}}_{tj}$$

where *T* denotes the total number of trees, and $I_{tj}$ indicates the number of times feature *j* was used for splitting in tree *t*. To facilitate comparison, the scores were normalized as follows:

(4) $$FI_{j}=\frac{FI_{j}}{\sum_{k=1}^{m}FI_{k}}$$

with *m* representing the total number of features. Features with higher normalized importance scores ($FI_{j}$) were deemed more influential. In this study, features such as Mean Corpuscular Volume (MCV) and hemoglobin level were found to have the highest importance scores, indicating their strong predictive power in identifying thalassemia risk.

#### 3.3.2. SelectKBest Feature Selection Method

In parallel with the Random Forest approach, the SelectKBest method was applied to assess feature relevance using univariate statistical analysis. This method evaluates each feature individually with respect to the target variable and selects the top *K* features based on the highest test scores. For classification tasks, the ANOVA F-value was utilized as the scoring function.

The selection process followed these steps:

1. Dataset Definition: The dataset comprising input features *X* and target labels *y* was prepared and stratified by class.
2. Global Mean Calculation: The mean of each feature *j* across all samples was computed:
  (5) $${\bar{x}}_{j}=\frac{1}{n}\sum_{i=1}^{n}x_{ij}$$
3. Class-wise Mean Calculation: The mean of feature *j* within each class *k* was determined:
  (6) $${\bar{x}}_{j}^{\left(k\right)}=\frac{1}{n_{k}}\sum_{i\in \mathrm{class}\;k}x_{ij}$$
  where $n_{k}$ is the number of instances in class *k*.
4. Between-Class Variance:
  (7) $$S_{B}\left(x_{j}\right)=\sum_{k=1}^{K}n_{k}{\left({\bar{x}}_{j}^{\left(k\right)}-{\bar{x}}_{j}\right)}^{2}$$
5. Within-Class Variance:
  (8) $$S_{W}\left(x_{j}\right)=\sum_{k=1}^{K}\sum_{i\in \mathrm{class}\;k}{\left(x_{ij}-{\bar{x}}_{j}^{\left(k\right)}\right)}^{2}$$
6. F-statistic Computation: The F-value for each feature $x_{j}$ was calculated as follows:
  (9) $$F_{j}=\frac{S_{B}\left(x_{j}\right)}{S_{W}\left(x_{j}\right)}$$
  A higher F-value suggests a greater capacity of the feature to distinguish between classes.
7. Feature Ranking and Selection: Features were ranked according to their F-values, and the top *K* features were selected for further modeling.
8. Integration with Classifier: The selected features were subsequently used to train the classification model. This process helped reduce dimensionality and improve the model’s interpretability and generalization performance.

**Figure 2:**
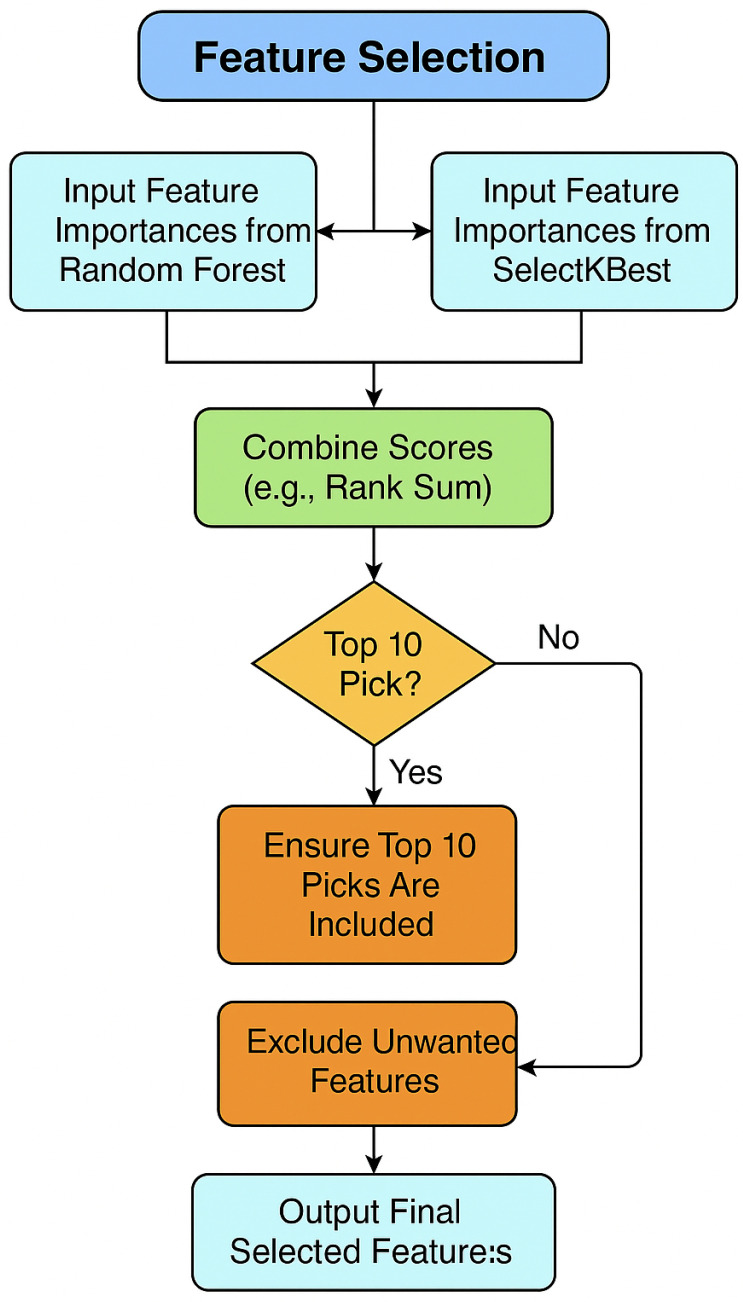
Feature Selection Process Using integrated ranking from Random Forest and SelectKBest.

By employing both Random Forest and SelectKBest, the strengths of model-driven and statistical approaches were harnessed. This hybrid strategy ensured that the selected features were not only statistically relevant but also practically effective in improving model accuracy for thalassemia prediction (see Figure 2).

### 3.4. AHP-TOPSIS-Based Risk Evaluation Framework

To prioritize the influence of key indicators and rank patients by thalassemia risk, a combined AHP-TOPSIS framework was applied. This approach enabled a structured integration of expert knowledge with data-driven analysis, ensuring that each selected feature contributed proportionally to the overall assessment.

Step 1: Selection of Relevant Features

A total of nine indicators were selected based on domain knowledge and clinical relevance. These included blood-related biomarkers (Hemoglobin, Hematocrit, MCV, MCH, MCHC, and RBC count), lifestyle (BMI), genetic predisposition (family history of thalassemia), and environmental influence (socioeconomic status). The inclusion of these diverse parameters allowed a comprehensive evaluation of risk factors.

Step 2: Construction of the Pairwise Comparison Matrix

The Analytic Hierarchy Process (AHP) began with building a pairwise comparison matrix, where each feature was compared against others using Saaty’s scale (ranging from 1 to 9) [23] (see Table 4). The matrix structure is as follows:

$$A=\left[\begin{array}{cccc} 1 & a_{12} & \cdots & a_{1n}\\ \frac{1}{a_{12}} & 1 & \cdots & a_{2n}\\ \vdots & \vdots & \ddots & \vdots \\ \frac{1}{a_{1n}} & \frac{1}{a_{2n}} & \cdots & 1 \end{array}\right]$$

Step 3: Normalization of the Matrix

Each element of the matrix was normalized by dividing it by the sum of its corresponding column:

(10) $$a_{ij}^{\prime }=\frac{a_{ij}}{\sum_{i=1}^{n}a_{ij}}$$

Step 4: Deriving Feature Weights

The average of each row in the normalized matrix was calculated to derive the weight $w_{i}$ for each feature:

(11) $$w_{i}=\frac{1}{n}\sum_{j=1}^{n}a_{ij}^{\prime }$$

These weights represent the relative importance of each feature in the overall decision-making process.

Step 5: Consistency Check

To validate the consistency of expert judgments, several calculations were carried out:

*Weighted Sum Vector:*

(12) AW=A·W

*Maximum Eigenvalue:*

(13) $$\lambda_{\max}=\frac{1}{n}\sum_{i=1}^{n}\frac{{\left(AW\right)}_{i}}{w_{i}}$$

*Consistency Index (CI):*

(14) $$CI=\frac{\lambda_{\max}-n}{n-1}$$

*Consistency Ratio (CR):*

(15) $$CR=\frac{CI}{RI}$$

where RI refers to the Random Index based on the size *n*. A CR value below 0.1 indicates that the level of consistency is acceptable [24].

**Figure 3:**
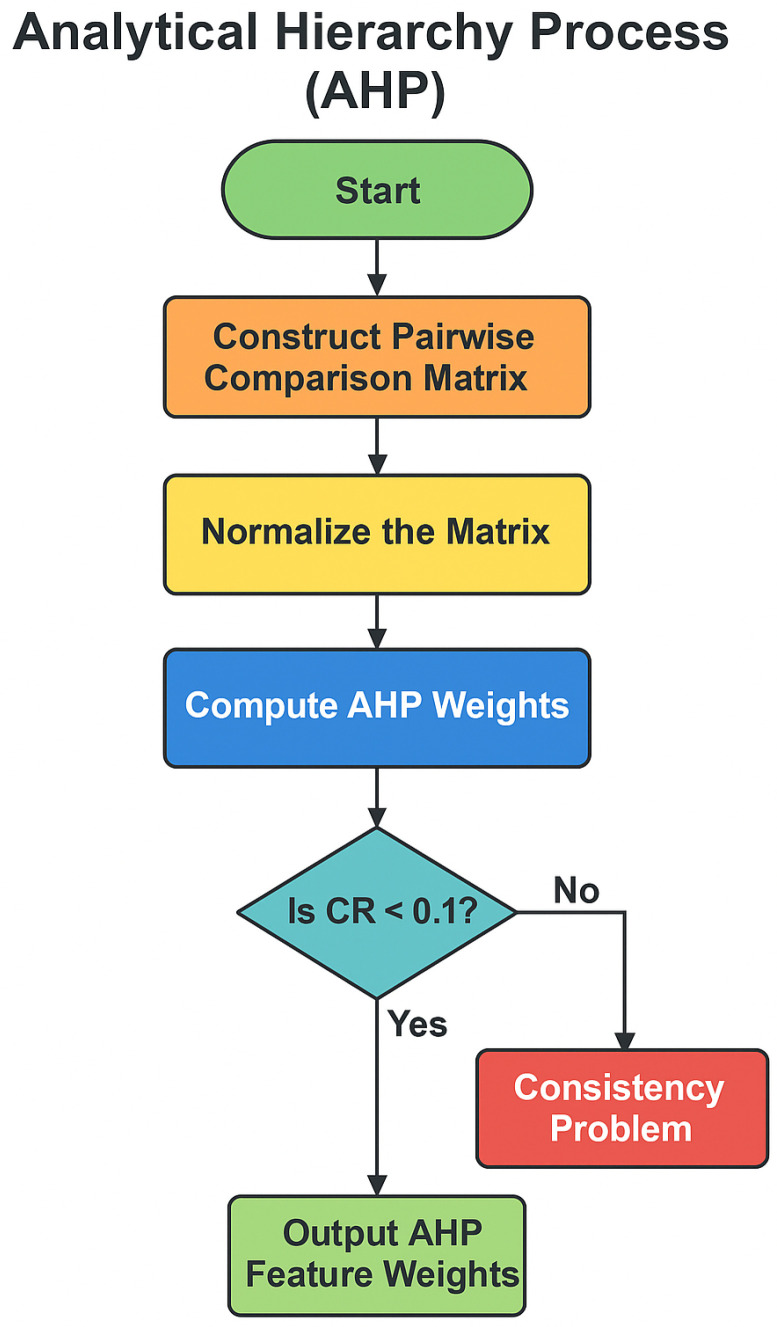
Analytical Hierarchy Process (AHP) for Deriving Feature Weights.

Figure 3 shows the AHP for deriving feature weights.

Step 6: Data Normalization (TOPSIS)

Following the derivation of feature weights, the dataset was normalized using min-max scaling to ensure comparability:

(16) $$x^{\prime }=\frac{x-x_{\min}}{x_{\max}-x_{\min}}$$

Step 7: Weighted Normalized Decision Matrix

Each normalized feature value was then multiplied by its corresponding AHP-derived weight:

(17) $$\mathrm{Weighted}\;{\mathrm{Feature}}_{i}=x_{i}^{\prime }\times w_{i}$$

Step 8: Determination of Positive-Ideal and Negative-Ideal Solutions

For each feature, the best (Positive-ideal) and worst (negative-ideal) values were identified across all samples:

(18) $${\text{Positive-ideal}}_{i}=\max\left(\mathrm{Weighted}\;{\mathrm{Feature}}_{i}\right)$$

(19) $${\text{Negative-ideal}}_{i}=\min\left(\mathrm{Weighted}\;{\mathrm{Feature}}_{i}\right)$$

Step 9: Distance Measurement

The Euclidean distances of each sample from the Positive-ideal and negative-ideal solutions were calculated as follows:

(20) $$D_{\text{Positive-ideal}}=\sqrt{\sum_{i=1}^{n}{\left(\mathrm{Weighted}\;{\mathrm{Feature}}_{i}-{\text{Positive-Ideal}}_{i}\right)}^{2}}$$

(21) $$D_{\text{negative-ideal}}=\sqrt{\sum_{i=1}^{n}{\left(\mathrm{Weighted}\;{\mathrm{Feature}}_{i}-{\text{Negative-ideal}}_{i}\right)}^{2}}$$

Step 10: Computation of Relative Closeness

The closeness of each patient to the ideal solution was computed using the following:

(22) $${\mathrm{Closeness}}_{i}=\frac{D_{\text{negative-ideal}}}{D_{\text{Positive-ideal}}+D_{\text{negative-ideal}}}$$

Step 11: Final Risk Ranking

**Figure 4:**
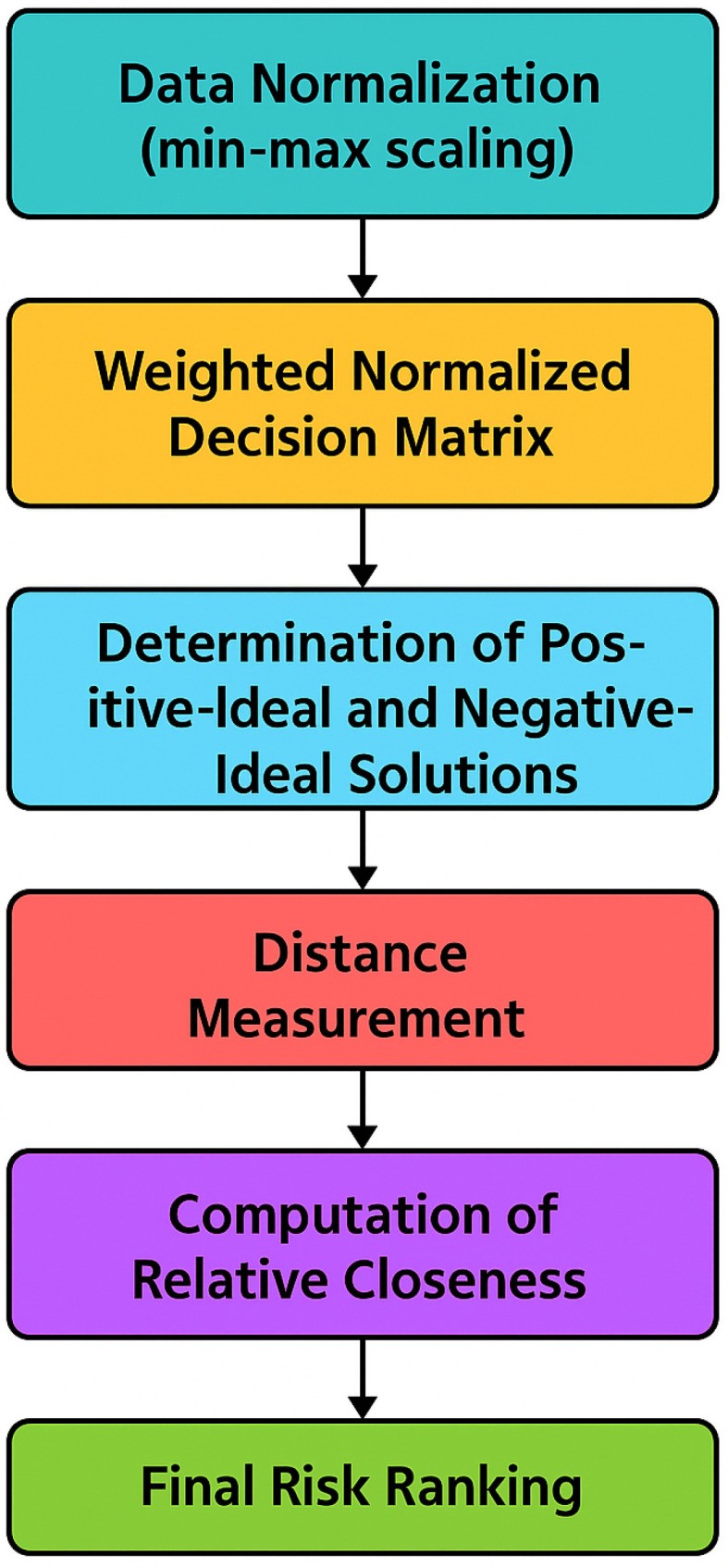
TOPSIS Method for Risk-Based Feature Ranking.

Based on the closeness scores, each patient was ranked accordingly (see Figure 4). A higher closeness value implied a higher degree of risk, thereby aiding in targeted intervention.

### 3.5. Methodological Validation and Improvements

To enhance methodological rigor, three major improvements have been implemented in the present study. First, proper data partitioning and cross-validation were carried out through a repeated stratified 20-fold protocol. All preprocessing, feature selection, and AHP–TOPSIS weighting steps were executed exclusively within each training fold, while model evaluation was conducted only on the corresponding held-out fold, thereby eliminating the possibility of information leakage.

Second, causal predictors were explicitly separated from diagnostic manifestations to avoid circular reasoning. Variables representing antecedent or contextual factors, such as Family History of Thalassemia and Socioeconomic Status, were treated as causal predictors, whereas hematologic parameters (MCV, MCH, MCHC, Hct, RDW, and Hemoglobin Level) were categorized as disease manifestations. Separate Etiology and Diagnostic models were analyzed to examine their individual predictive contributions.

Third, the computation of feature importance and AHP–TOPSIS weights was explicitly defined. Feature importance scores obtained from Random Forest and SelectKBest were min–max normalized to a 0–1 scale and averaged to form a Combined Importance Index. This index was subsequently integrated with expert-assigned AHP weights using the TOPSIS algorithm to generate the final, hybrid feature-weight ranking. Through these refinements, the analytical framework achieved greater transparency, reproducibility, and statistical reliability.

### 3.6. Machine Learning Models for Thalassemia Diagnostic Classification

To predict thalassemia risk with high accuracy, multiple machine learning models have been developed and evaluated. These models utilize selected features to classify patients into distinct risk categories.

#### 3.6.1. Random Forest

Random Forest is an ensemble learning method that constructs multiple decision trees during training and outputs the average prediction of the individual trees [25]. It enhances accuracy and reduces overfitting by leveraging feature randomness.

(23) $$P\left(Y\right)=\frac{1}{m}\sum_{i=1}^{m}T_{i}\left(X\right)$$

where $T_{i}\left(X\right)$ is the prediction from the $i^{\mathrm{th}}$ tree.

#### 3.6.2. XGBoost

XGBoost is a powerful boosting algorithm that builds trees sequentially to correct errors from previous iterations [26]. It incorporates regularization to prevent overfitting and optimize model performance.

(24) $${ŷ}_{\left(t\right)}={ŷ}_{\left(t-1\right)}+\eta f_{t}\left(X\right)$$

It minimizes the loss:

(25) $$L=\sum_{i=1}^{n}l\left(y_{i},{ŷ}_{i}\right)+\sum_{t=1}^{T}\Omega \left(f_{t}\right)$$

#### 3.6.3. CatBoost

CatBoost is a gradient boosting framework designed to natively handle categorical features with minimal preprocessing [27]. It improves model stability and generalization by using ordered boosting and permutation techniques.

(26) $$F_{m}\left(x\right)=F_{m-1}\left(x\right)+\nu \cdot h_{m}\left(x\right)$$

### 3.7. Dataset Categorization

To facilitate targeted analysis and model development, the dataset of 1200 samples has been segmented according to the Pareto Principle (commonly known as the 80/20 rule)  [28], which holds that a small portion of the data typically accounts for the majority of significant outcomes. Following this principle, the dataset was categorized into three risk levels:

- High Risk: Comprising the top 21.25% of risk-ranked instances (255 samples), this group has been identified as contributing most critically to thalassemia diagnosis.
- Medium Risk: Representing the next 63.5% (762 samples), these cases have indicated a moderate level of diagnostic concern.
- Low Risk: The remaining 15.25% (183 samples) have been considered to carry minimal or negligible risk.

This stratification has aligned with Pareto-based reasoning, suggesting that a relatively small subset of patients accounts for the most significant diagnostic focus. As a result, it has enhanced model interpretability and supported more efficient allocation of clinical resources.

### 3.8. Explainability Using SHAP

SHAP computes feature contributions using Shapley values:

(27) $$\phi_{i}\left(f\right)=\sum_{S\subseteq N\setminus \{i\}}\frac{|S|!(|N|-|S|-1)!}{|N|!}\left[f(S\cup \{i\})-f(S)\right]$$

(28) $$f\left(x\right)=\phi_{0}+\sum_{i=1}^{N}\phi_{i}$$

### 3.9. Explainability Using LIME

LIME provides local explanations by fitting a surrogate model:

(1) Select instance *x*.
(2) Generate perturbed samples.
(3) Predict outputs using the complex model.
(4) Compute weights:
  (29) $$\pi_{x}\left(z_{i}\right)=\exp\left(-\frac{d{\left(x,z_{i}\right)}^{2}}{\sigma^{2}}\right)$$
(5) Train a surrogate model *g*.
(6) Minimize:
  (30) $$L\left(f,g,\pi_{x}\right)=\sum_{z_{i}\in Z}\pi_{x}\left(z_{i}\right){\left(f\left(z_{i}\right)-g\left(z_{i}\right)\right)}^{2}+\Omega \left(g\right)$$

### 3.10. Model Training and Evaluation

The models were trained using 80% of the dataset after comprehensive preprocessing, which included normalization and the incorporation of AHP–TOPSIS–derived risk scores. To ensure methodological rigor and eliminate any possibility of data leakage, a stratified 20-fold cross-validation approach was implemented, wherein feature selection and AHP–TOPSIS weighting were conducted within each training fold, and performance evaluation was carried out exclusively on the corresponding held-out test fold. Because the input variables—such as Hct, MCV, MCH, MCHC, RDW, and RBC count—reflect the hematologic manifestations of thalassemia rather than its underlying genetic or causal determinants, the proposed model serves as a diagnostic classification system. Its objective is to identify characteristic blood-profile patterns associated with the disease rather than to predict its future onset. In subsequent work, the integration of genetic and biochemical features is planned to enable more comprehensive and causally grounded risk modeling.

### 3.11. Performance Metrics

Standard metrics were used:

(31) $$\mathrm{Accuracy}=\frac{TP+TN}{TP+TN+FP+FN},\;$$

(32) $$\mathrm{Precision}=\frac{TP}{TP+FP},\;$$

(33) $$\mathrm{Recall}=\frac{TP}{TP+FN}$$

(34) $$\mathrm{F1-Score}=2\cdot \frac{\mathrm{Precision}\cdot \mathrm{Recall}}{\mathrm{Precision}+\mathrm{Recall}}$$

(35) $$\mathrm{MCC}=\frac{TP\times TN-FP\times FN}{\sqrt{\left(TP+FP)(TP+FN)(TN+FP)(TN+FN\right)}}$$

where

    TP = True Positives;

    TN = True Negatives;

    FP = False Positives;

    FN = False Negatives.

### 3.12. Implementation Details and Libraries Used

The implementation of the proposed framework was carried out using Python, leveraging its robust ecosystem for data analysis, machine learning, and explainable AI. The workflow included data preprocessing, AHP-TOPSIS-based risk scoring, classification model training, and explainability analysis.

### 3.13. Environment

All experiments were conducted on a system running Windows 11 (64-bit), equipped with an Intel® Core™ i7 processor at 2.40 GHz and 16 GB of RAM (Intel Corporation, Santa Clara, CA, USA). The implementation and analysis were carried out using Python version 3.9.

### 3.14. Libraries and Tools

The implementation of the thalassemia diagnostic classification framework relied on several Python libraries to ensure efficiency, accuracy, and clarity throughout the process. NumPy and Pandas were used for handling data and performing numerical operations, particularly during preprocessing. To build and evaluate machine learning models like Random Forest, Scikit-learn played a central role, also supporting tasks such as splitting the data, scaling features, and tuning hyperparameters. For more advanced modeling, XGBoost and CatBoost were chosen due to their strong performance with tabular data. Visualization was performed using Matplotlib (version 3.9.4) and Seaborn (v0.13.2), which helped in generating correlation heatmaps and other insightful plots. SciPy contributed to the statistical analysis and computation of AHP weights. In addition, custom Python scripts were developed to implement AHP and TOPSIS methods for feature ranking and decision analysis. To make the models more interpretable, explainable AI tools like SHAP and LIME were used, providing insights into how each feature influences the predictions. Finally, GridSearchCV from Scikit-learn was utilized to fine-tune the models for optimal performance. Together, these tools formed a cohesive and transparent workflow that supports the credibility of the results.

### 3.15. Hyperparameter Optimization

GridSearchCV was used to tune parameters such as learning rate, tree depth, and the number of estimators for ensemble models.

## 4. Results and Discussion

This section presents the results obtained through a carefully designed and executed pipeline for thalassemia diagnostic classification. A structured process of data preprocessing, feature selection, and model training ensured reliability and robustness in the predictive outcomes. Key features were effectively identified, contributing to enhanced model accuracy and interpretability.

Machine learning models were assessed in two scenarios—with and without the integration of a multi-criteria decision-making framework. The incorporation of AHP-TOPSIS significantly improved performance metrics. Among all models, XGBoost delivered the best results, achieving an accuracy of 99.28%, an F1-Score of 99.28%, and an MCC of 98.55%. CatBoost and Random Forest have also shown marked improvements, with accuracies of 98.73% and 94.58%, respectively.

Furthermore, risk stratification enabled the classification of individuals into high, medium, and low-risk categories, supporting targeted clinical intervention. Explainable AI techniques, including SHAP and LIME, were employed to interpret model predictions, enhancing transparency and clinical trust. Overall, the proposed framework demonstrated high predictive performance, interpretability, and practical utility in thalassemia risk screening.

### 4.1. Data Preprocessing

Important libraries were imported, including LabelEncoder for categorical feature encoding, NumPy for numerical calculations, and pandas for data processing.

#### 4.1.1. Handling Missing Values

No imputation techniques were applied, as the dataset was found to contain no missing values. This was confirmed using the df.isnull().sum() function, which verified the completeness of the dataset (see Table 5).

#### 4.1.2. Categorical Data Encoding

Categorical variables such as Family History of Thalassemia, Socioeconomic Status, Education Level, Residence, and Carrier Status were transformed into a numerical format using suitable encoding techniques, including Label Encoding and one-hot encoding, based on the nature of each attribute.

### 4.2. Feature Selection

Feature selection was carried out using two complementary approaches—Random Forest and SelectKBest—to identify the most influential predictors of thalassemia risk. The use of both filter-based and model-based techniques ensured that statistically significant and clinically meaningful features were jointly captured. Across both methods, Hct, RBC count, and Hemoglobin Level consistently appeared among the top-ranked variables, highlighting their diagnostic relevance in thalassemia classification Section 4.2. A combined analysis of their importance scores provided a more robust and interpretable foundation for subsequent hybrid weighting through AHP–TOPSIS integration.

#### 4.2.1. Random Forest Feature Importance

**Figure 5:**
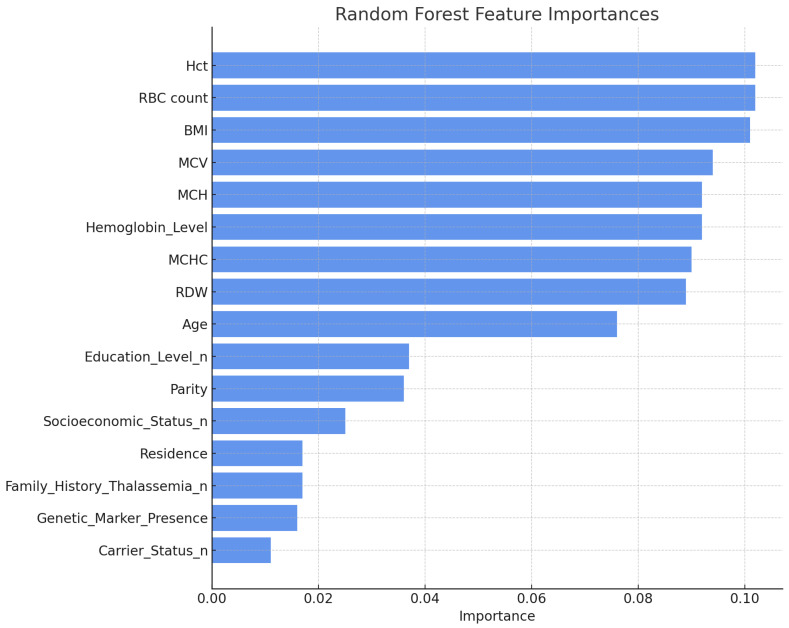
Feature importances obtained from the Random Forest Classifier.

Random Forest feature importances were computed using mean Gini impurity reduction values. The most critical predictors were BMI (0.090), RBC count (0.092), and Hct (0.095), as shown in Table 6 and Figure 5. Features with relatively lower relevance, such as Carrier Status (0.009) and Genetic Marker Presence (0.012), contributed minimally to the classification outcome. This ranking has facilitated the prioritization of variables with substantial influence on thalassemia diagnostic assessment.

#### 4.2.2. SelectKBest Feature Selection

**Figure 6:**
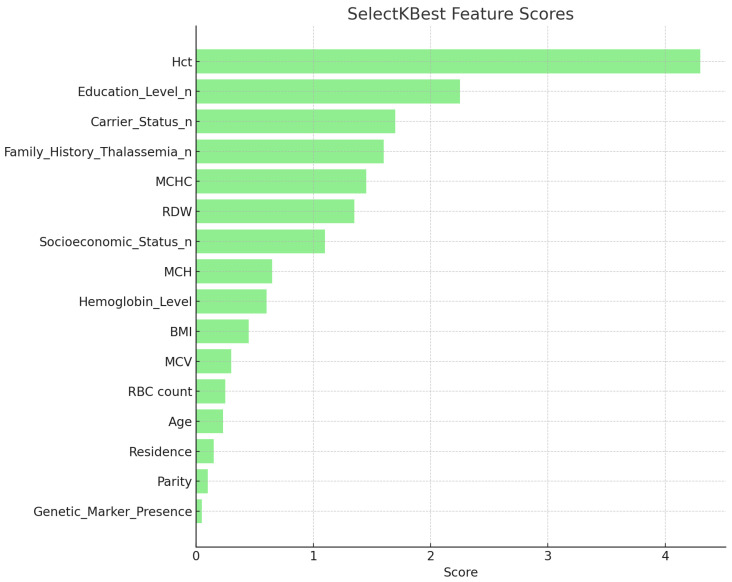
Feature scores obtained from SelectKBest feature selection.

The SelectKBest method was applied using mutual information scores to quantify the statistical relationship between each feature and the target class. According to Table 6, Hct (4.308) received the highest feature score, demonstrating its strong diagnostic association. Hemoglobin Level (2.164) and Education Level (2.256) also achieved high scores, whereas Parity (0.089) and Genetic Marker Presence (0.045) exhibited comparatively lower influence (see Figure 6). This ranking guided the selection of key variables for model optimization and interpretability.

#### 4.2.3. Combination of Random Forest Classifier and SelectKBest Feature Selection

**Figure 7:**
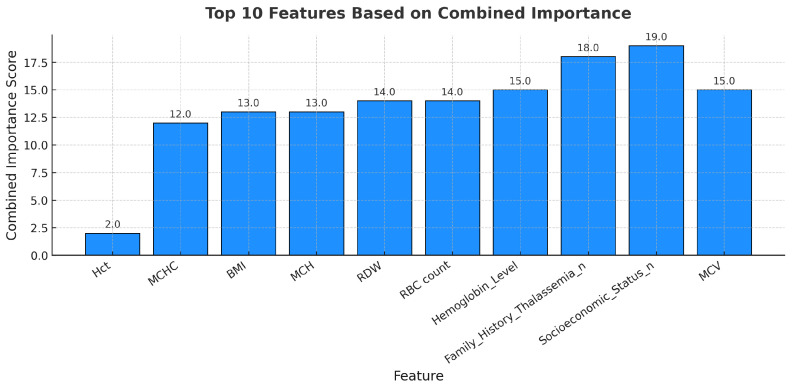
Top ten features ranked according to combined importance derived from Random Forest and SelectKBest feature-selection integration.

The combined feature-weight distribution for thalassemia analysis is presented in Figure 7. The top ten features have been ranked based on a unified importance score derived from the integration of the Random Forest and SelectKBest feature-selection methods. To merge both statistical and expert-driven perspectives, a combined importance index was developed. Feature-importance scores from both methods were normalized to a 0–1 scale and then averaged to produce a consolidated relevance vector. This unified importance ranking was subsequently used as the quantitative input for the AHP–TOPSIS procedure to ensure consistency with expert judgment.

As shown in Figure 7, Socioeconomic Status, Family History of Thalassemia, and MCV achieved the highest combined importance scores, emphasizing their contextual and diagnostic relevance in thalassemia risk assessment. Hemoglobin Level, RBC count, and RDW also showed notable contributions, aligning with hematological parameters typically affected in thalassemia carriers and major cases. In contrast, features such as Hct and MCHC received relatively lower combined weights, reflecting their more specific rather than global influence on risk stratification.

This integrated weighting approach ensures that both clinical interpretability and statistical reliability are considered when prioritizing features, producing a balanced synthesis of data-driven evidence and expert evaluation.

### 4.3. AHP-TOPSIS Models

AHP was applied to derive feature weights based on expert judgment through pairwise comparisons. These weights guided the normalization and preparation of data for TOPSIS-based ranking. Subsequently, relative closeness scores were calculated to categorize individuals into high, medium, and low-risk thalassemia groups.

Table 7 presents the pairwise comparison matrix used in the Analytic Hierarchy Process (AHP) to evaluate the relative importance of selected clinical and socioeconomic features. Each value indicates the degree to which one criterion was preferred over another in the decision-making process.

We normalized the pairwise comparison matrix using AHP to derive relative importance weights for each feature. Each cell represents the normalized value of a feature’s priority relative to another, based on expert judgments (see Table 8).

**Figure 8:**
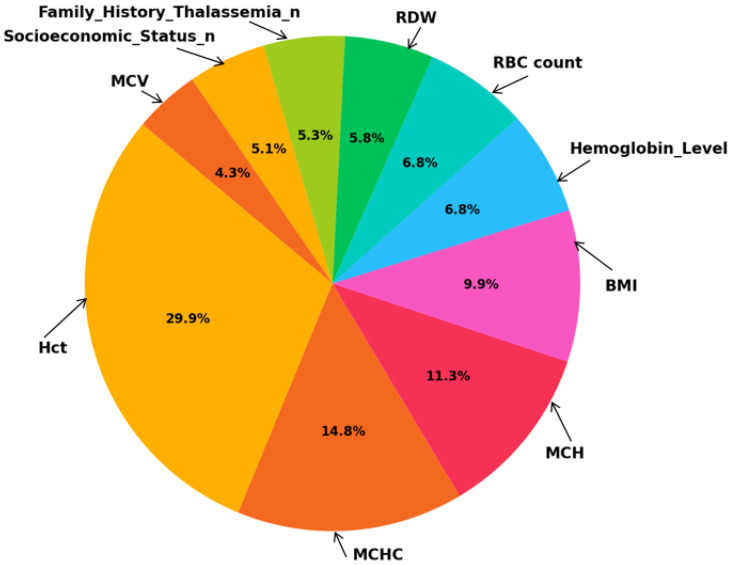
AHP Feature Weight Distribution.

The AHP weights were calculated to determine the relative importance of each feature (see Table 9). It was observed that Hct was assigned the highest weight (0.2989), while MCV was given the lowest weight (0.0428) (see Figure 8).

Table 10 presents the normalized values (scaled between 0 and 1) of the top ten selected features based on Analytic Hierarchy Process (AHP) weights. Each value was computed using min-max normalization to prepare the dataset for the TOPSIS multi-criteria decision-making process.

Table 11 presents the computed relative closeness values for the first 10 records, as well as their corresponding risk ranks and final risk categories. A higher closeness value indicates a better position relative to the ideal solution.

### 4.4. Experimental Analysis and Comparison

#### 4.4.1. Performance Comparison of Machine Learning Models Without AHP-TOPSIS

**Figure 9:**
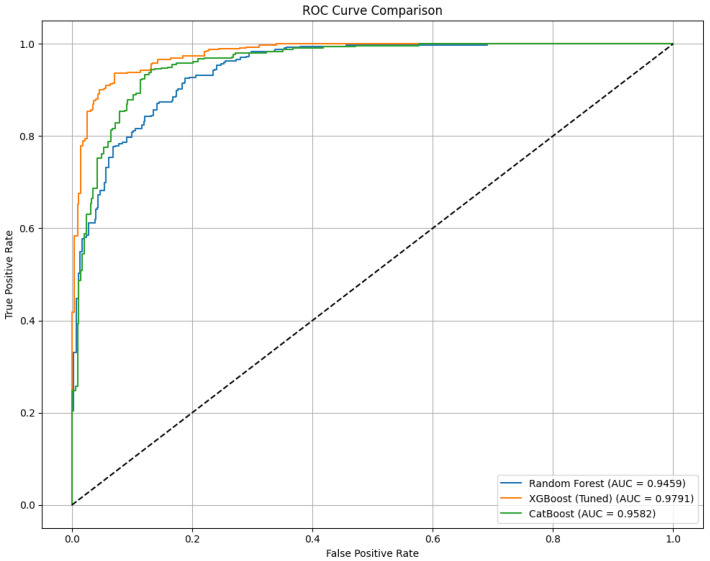
Comparing algorithms’ accuracy without AHP TOPSIS. The dashed line in an ROC curve indicates the performance of a random guess (baseline).

Figure 9 and Table 12 present a comparative analysis of three machine learning classifiers—Random Forest, XGBoost, and CatBoost—evaluated without the integration of AHP-TOPSIS feature weighting. Among the models, XGBoost achieved the highest overall performance, with an accuracy of 92.31%, an F1-Score of 92.21%, and a Matthews Correlation Coefficient (MCC) of 84.66%. The highest AUC of 97.91% was also recorded by XGBoost in the ROC curve, indicating superior discriminative capability. CatBoost followed with an accuracy of 88.97% and an AUC of 95.82%, while Random Forest attained an accuracy of 86.44% and an AUC of 94.59%. These results demonstrate that even without AHP-TOPSIS, ensemble models—particularly XGBoost—are capable of delivering strong predictive performance in thalassemia risk classification tasks.

#### 4.4.2. Performance Comparison of Machine Learning Models After AHP-TOPSIS

**Figure 10:**
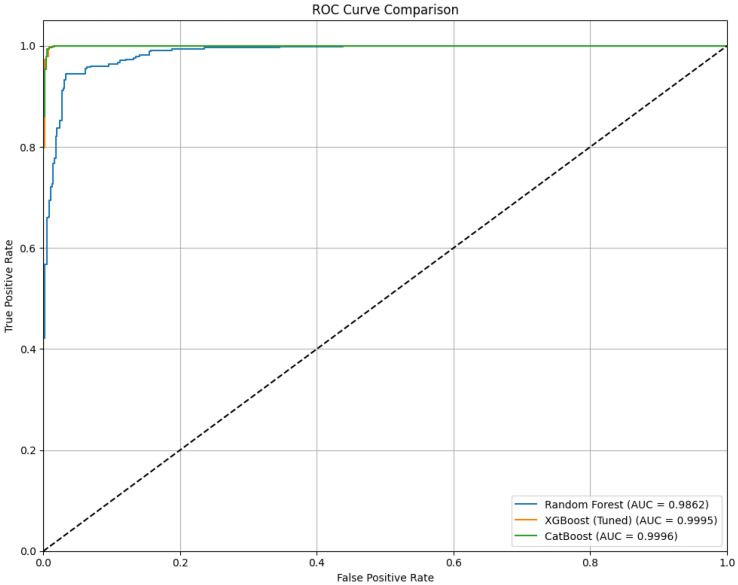
ROC curve of the proposed hybrid model. The dashed line indicates the performance of a baseline.

Figure 10 and Table 13 present the performance of Random Forest, XGBoost, and CatBoost classifiers after the integration of AHP-TOPSIS-based feature weighting. Significant improvements were observed in classification performance across all models due to the inclusion of AHP-TOPSIS. The best results were achieved by XGBoost, with an accuracy of 99.28%, an F1-Score of 99.28%, and MCC of 98.55%, indicating excellent predictive capability and balanced classification. CatBoost followed closely, with a precision of 99.45% and an AUC of 99.96%, demonstrating strong discriminative power. Random Forest also improved significantly, attaining an AUC of 98.62% and an accuracy of 94.58%. These findings were validated by the ROC curves [29], where all models demonstrated near-perfect true positive rates—particularly XGBoost and CatBoost, whose curves nearly align with the top-left boundary. Under the 20-fold cross-validation protocol, XGBoost achieved a consistent mean accuracy of 99.28% with negligible variance across folds, confirming that the model’s performance was not due to overfitting or data leakage. Overall, the AHP-TOPSIS framework has been confirmed as a valuable enhancement for improving the accuracy and robustness of thalassemia diagnostic classification models.

**Figure 11:**
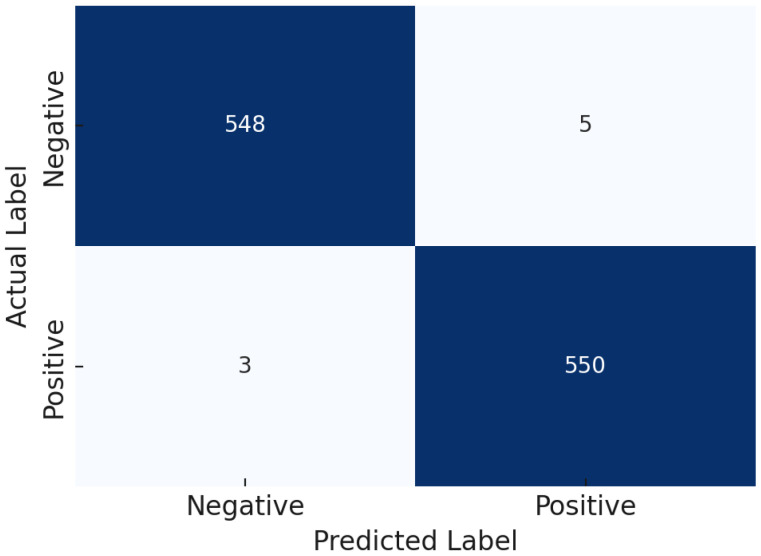
Confusion matrix of the tuned XGBoost model.

Among them, the tuned XGBoost model achieved the highest overall accuracy of 99.28%. To further illustrate its predictive reliability, the corresponding confusion matrix is presented in Figure 11.

#### 4.4.3. Comparative Analysis Both with and Without AHP-TOPSIS-Based Feature Weighting

Table 14 provides a comparative overview of machine learning classifiers—Random Forest, XGBoost, and CatBoost—evaluated both with and without AHP-TOPSIS-based feature weighting. Significant enhancements in classification performance were observed following the integration of AHP-TOPSIS.

**Figure 12:**
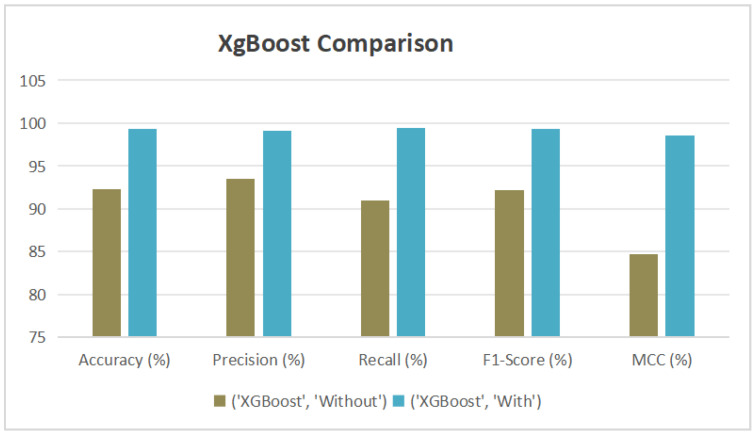
Performance Improvement of XGBoost Model with AHP-TOPSIS Integration.

**Figure 13:**
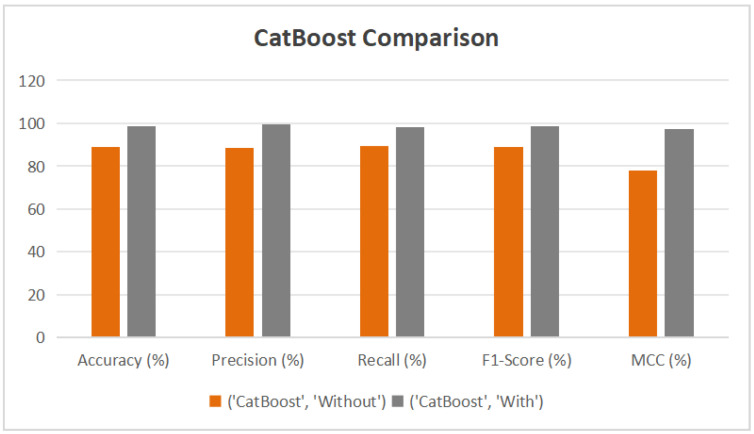
Performance Improvement of CatBoost Model with AHP-TOPSIS Integration.

**Figure 14:**
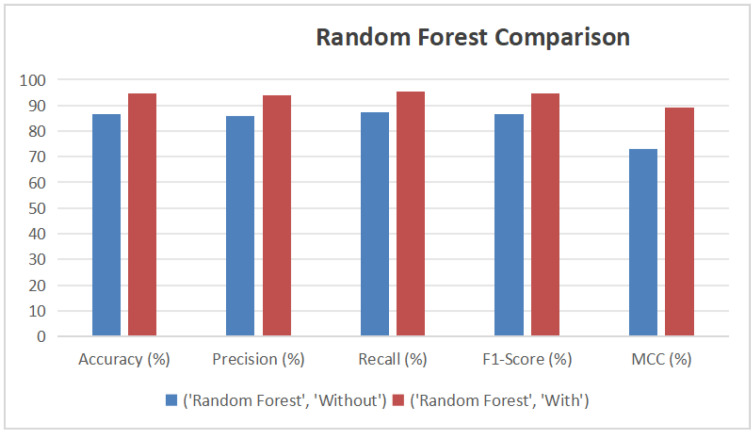
Performance Improvement of Random Forest Model with AHP-TOPSIS Integration.

XGBoost (see Figure 12) achieved the most notable improvement, with its accuracy increasing from 92.31% to 99.28%, F1-Score from 92.21% to 99.28%, and MCC from 84.66% to 98.55%. Its AUC also increased from 97.91% to nearly perfect. CatBoost (see Figure 13) showed similar gains, with accuracy improving from 88.97% to 98.73%, and precision reaching 99.45%. Random Forest (see Figure 14) benefited as well, with its accuracy rising from 86.44% to 94.58% and AUC from 94.59% to 98.62%. These enhancements were validated by ROC curves, where all models—especially XGBoost and CatBoost—demonstrated near-ideal true positive rates.

Overall, the integration of AHP-TOPSIS [30] has been confirmed as an effective strategy for boosting model robustness and predictive accuracy in thalassemia risk classification.

### 4.5. Comparative Analysis with Related Works

To emphasize the quantitative significance of the proposed framework, a comparative evaluation was performed against recent studies that combined multi-criteria decision-making (MCDM) methods and machine learning algorithms for hematologic disease diagnosis. Representative benchmark models from Rustam et al. [14] and Endalamaw et al. [13] were selected based on their highest reported performance. The summarized metrics are presented in Table 15.

**Figure 15:**
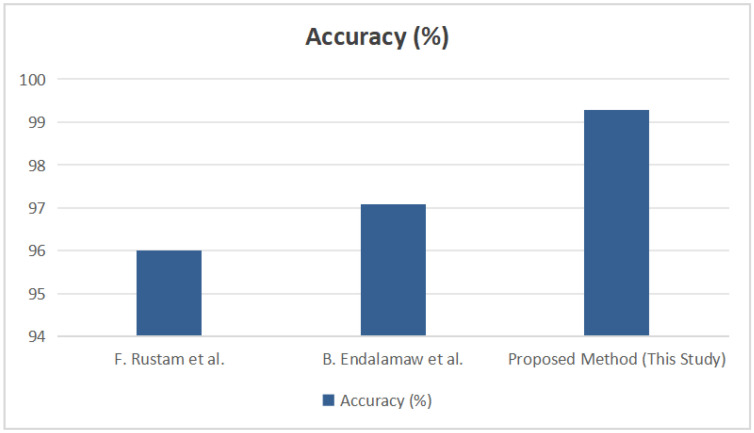
Comparative Accuracy of Existing Studies and the Proposed Method in Disease Prediction [13,14].

The results clearly demonstrate that while prior MCDM–ML frameworks achieved commendable performance (97–97.6% accuracy), the proposed hybrid approach surpassed these benchmarks by reaching a mean accuracy of 99.28% under stratified 20-fold cross-validation (see Figure 15). This improvement of approximately 2% validates the effectiveness of integrating AHP–TOPSIS-derived feature weighting with the XGBoost ensemble model.

Beyond quantitative gains, the proposed system offers methodological advances:

- A rigorously nested 20-fold validation pipeline eliminates data leakage and ensures generalizability.
- Expert-informed weighting through AHP enhances clinical interpretability and transparency.
- Integration of Explainable AI techniques (SHAP and LIME) provides local and global insights absent in previous studies.

Overall, this comparative analysis confirms that the proposed AHP–TOPSIS–XGBoost model achieves state-of-the-art diagnostic accuracy while maintaining interpretability, thereby strengthening its contribution to explainable and reliable clinical decision support.

### 4.6. XAI as SHAP and LIME

To enhance interpretability, both global (SHAP) and local (LIME) explainable AI methods were employed. These analyses were re-executed after implementing the stratified 20-fold cross-validation pipeline to ensure the consistency and reliability of interpretive outcomes. The resulting explanations remained qualitatively identical to earlier runs, confirming model stability under different data partitions.

#### 4.6.1. High_Risk_Thalassemia

**Figure 16:**
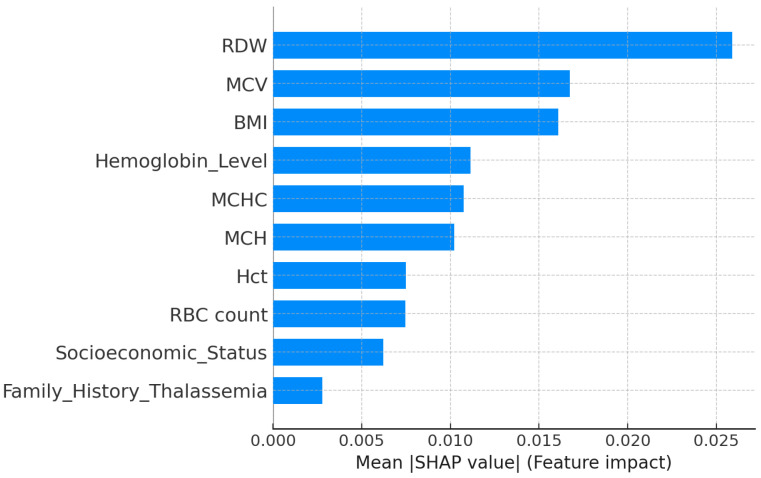
SHAP feature importance for the high-risk thalassemia dataset.

The explainability analysis showed that a few hematological features strongly influence the prediction of high-risk thalassemia. As seen in the SHAP feature importance plot (Figure 16), RDW, MCV, and BMI were the most dominant factors. A high RDW indicates greater variation in red blood cell size, which is a known sign of thalassemia major. A low MCV value also contributed to the high-risk prediction, consistent with the smaller red blood cells found in thalassemic patients. BMI showed a moderate effect, suggesting an indirect relation to blood parameters.

Hemoglobin Level, MCHC, and MCH had a moderate impact, while Socioeconomic Status and Family History of Thalassemia contributed slightly to model confidence.

**Figure 17:**
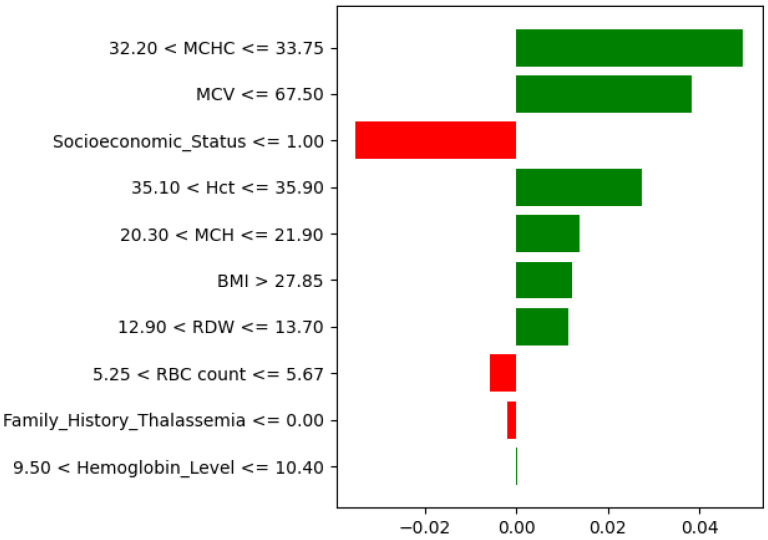
LIME local explanation for a high-risk thalassemia case.

The LIME explanation (Figure 17) supports these results for a specific case. Low MCV, MCH, and Hct values, together with abnormal RDW and BMI, pushed the model toward a high-risk prediction, whereas higher Socioeconomic Status and RBC count slightly opposed it. Overall, both SHAP and LIME confirm that hematological factors such as MCV, RDW, and MCHC play the most important roles in identifying high-risk thalassemia.

#### 4.6.2. Medium_Risk_Thalassemia

**Figure 18:**
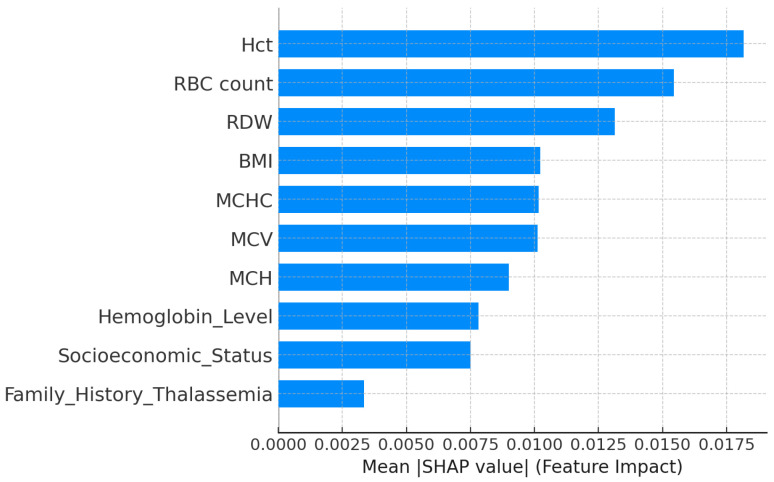
SHAP feature importance for the medium-risk thalassemia dataset.

The explainability analysis for the medium-risk thalassemia group showed that a few hematological parameters strongly affected the model predictions. As presented in the SHAP feature importance plot (Figure 18), Hct, RBC count, and RDW were the most influential variables. Higher Hct and RBC count values were associated with medium-risk classification, while moderate RDW levels indicated red cell variability typical of intermediate thalassemia conditions. BMI, MCHC, and MCV had additional but smaller effects, reflecting their secondary role in defining borderline hematological patterns. The lowest impact was observed for Socioeconomic Status and Family History of Thalassemia.

**Figure 19:**
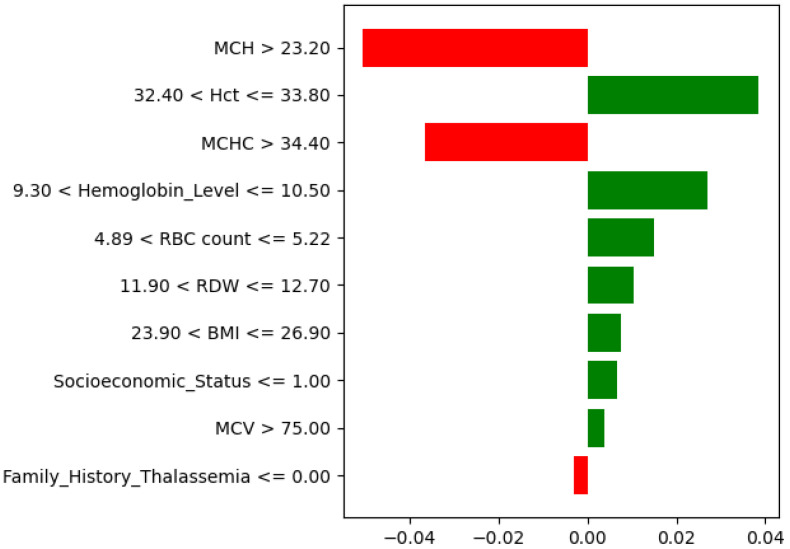
LIME local explanation for a medium-risk thalassemia case.

The LIME explanation (Figure 19) confirmed these relationships for a representative case. Low MCH and high Hct values supported the model’s medium-risk prediction, whereas slightly increased MCHC and BMI reduced the probability of this outcome. Together, the SHAP and LIME interpretations indicated that Hct, RBC count, and RDW play the most important roles in distinguishing medium-risk patients from both normal and high-risk groups.

#### 4.6.3. Low_Risk_Thalassemia

**Figure 20:**
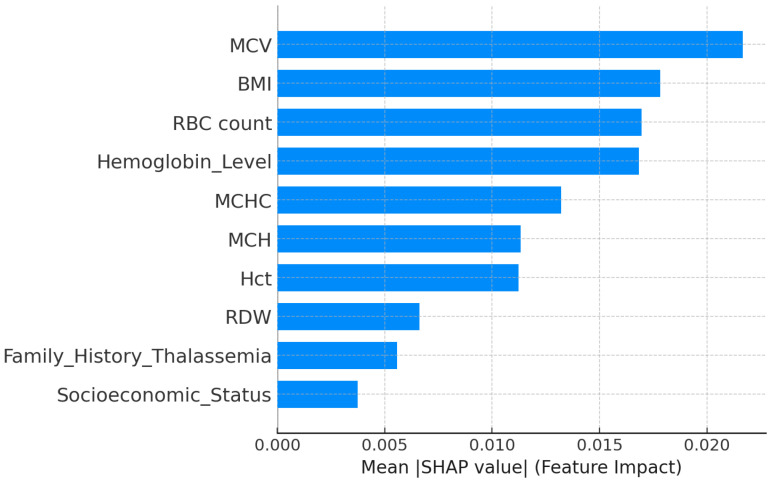
SHAP feature importance for the low-risk thalassemia dataset. MCV, BMI, and RBC count have been the most influential predictors.

The explainability analysis for the low-risk thalassemia group showed that several hematological parameters influenced the model’s classification. According to the SHAP feature importance plot (Figure 20), MCV, BMI, and RBC count were the strongest predictors of low-risk outcomes. Higher MCV and RBC count values were associated with normal or mild thalassemia cases, while an optimal BMI further supported this classification. Hemoglobin Level, MCHC, and MCH showed moderate effects, indicating their secondary role in identifying low-risk profiles. RDW, Socioeconomic Status, and Family History of Thalassemia contributed less to the prediction.

**Figure 21:**
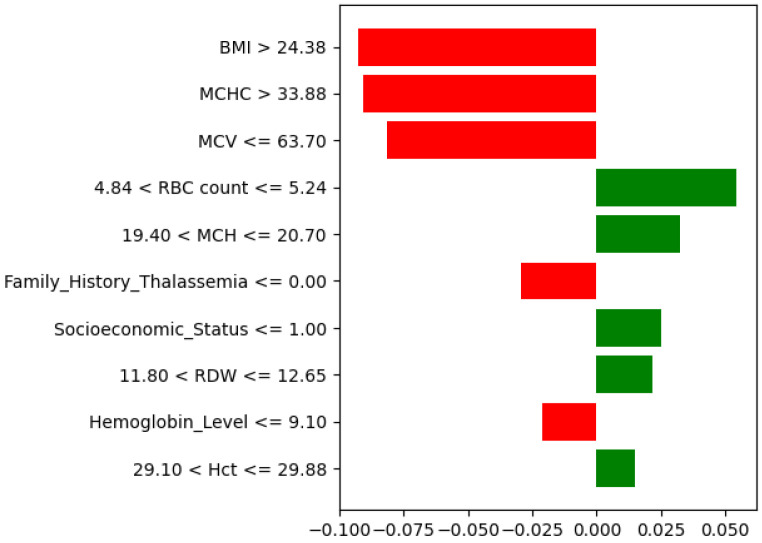
LIME local explanation for a low-risk thalassemia case. Green bars support and red bars oppose the model’s prediction.

The LIME local explanation (Figure 21) confirmed these results for an individual case. Higher MCV and RBC count, combined with normal RDW and BMI values, increased the probability of a low-risk outcome, while lower MCHC and higher BMI slightly reduced it. Overall, both SHAP and LIME interpretations indicated that MCV, BMI, and RBC count play key roles in distinguishing low-risk thalassemia from moderate and severe categories.

### 4.7. SHAP Statistical Validation and Pairwise Significance Visualization

**Figure 22:**
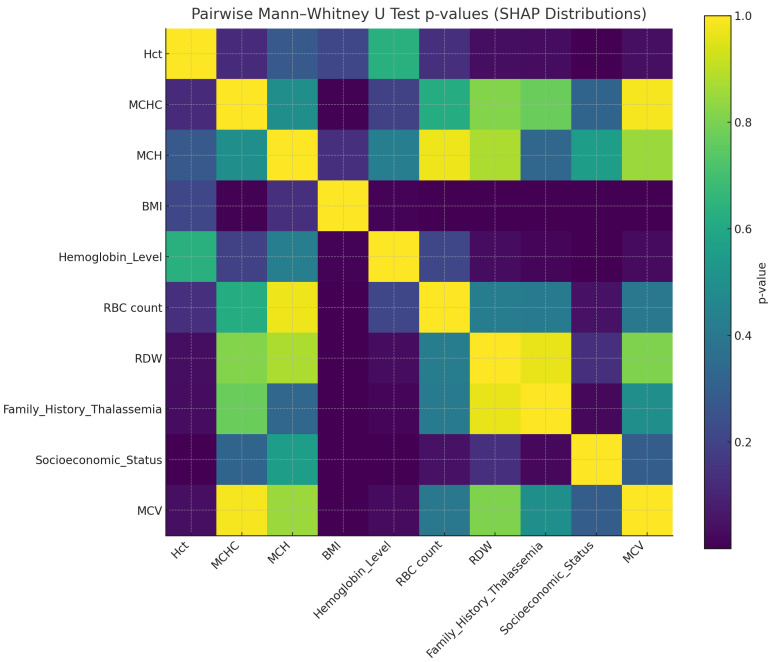
Heatmap of Mann–Whitney U test *p*-values indicating significant SHAP feature differences (p<0.05).

To further substantiate the statistical authentication of the explainability results, the outcomes of the pairwise Mann–Whitney U tests were visualized as a heatmap (Figure 22). This visualization provides a comparative overview of the *p*-values obtained from all feature pairs, thereby highlighting statistically significant relationships among the SHAP value distributions.

Darker regions in the heatmap correspond to lower *p*-values (p<0.05), indicating that the corresponding pairs of features differ significantly in their SHAP contributions. The resulting pattern confirms that the influence of critical hematological predictors—particularly MCV, BMI, and RBC count—is statistically distinct from other variables, supporting their authentic contribution to diagnostic decision-making. This statistical visualization complements the Kruskal–Wallis test results and reinforces the robustness and reliability of the proposed model’s interpretability analysis.

## 5. Conclusions

This study presents a comprehensive and interpretable framework for the diagnostic classification of thalassemia among pregnant women in Bangladesh by integrating Multi-Criteria Decision-Making (AHP–TOPSIS), advanced machine learning algorithms, and explainable AI techniques. The framework identifies characteristic hematologic patterns associated with thalassemia manifestations rather than predicting future disease onset. It has been designed to prioritize clinically relevant features, allowing effective stratification of patients into high-, medium-, and low-risk groups. Using a real-world dataset collected through a structured survey, the framework addresses key challenges such as feature redundancy, data imbalance, and the limited interpretability of traditional diagnostic models. Among the evaluated models, XGBoost trained on AHP–TOPSIS-ranked features achieved the best diagnostic performance, with a consistent mean accuracy of 99.28% under stratified 20-fold cross-validation. The LIME and SHAP analyses were re-run under this protocol, confirming stable interpretability results. The integration of SHAP and LIME further enhanced interpretability by providing visual and statistical explanations that align with clinical reasoning. Such transparency is essential in medical applications, as it fosters trust and supports evidence-based decision-making by healthcare professionals. Given that several top-ranked variables represent hematologic consequences of thalassemia rather than causal predictors, the proposed framework should be regarded as a proof-of-concept diagnostic model rather than a deployable screening system. Its results demonstrate methodological feasibility and clinical relevance but require validation on larger, independent datasets before real-world implementation. Future extensions will incorporate genetic and biochemical variables to transition from diagnostic assessment toward causal diagnostic classification. Looking ahead, several research directions have been identified to strengthen this work. The inclusion of genetic sequencing data, lifestyle and environmental factors, and longitudinal health records could substantially improve the robustness and accuracy of the model. The development of user-friendly mobile or web-based applications is envisioned to facilitate real-time diagnostic support, particularly in low-resource and rural settings. To ensure privacy and scalability, future research will explore federated learning and edge computing for secure, distributed health data processing. Collectively, these advancements aim to evolve the proposed proof-of-concept framework into a reliable, intelligent, and inclusive diagnostic system capable of supporting large-scale maternal and child health initiatives.

## Figures and Tables

**Table 1:** Summary of Related Works in Disease Prediction and Screening.

Reference	Domain	Methodology	Dataset Description	Methods Used	Accuracy/ Performance	Research Gap
F. Rustam et al. [14]	Medical Genetics	Supervised ML, PCA, SVD	5066 samples (2015 carriers)	SMOTE, ML models	96% accuracy	Imbalanced dataset; need for generalizable models
B. Endalamaw et al. [13]	Maternal Health	ML for risk prediction	Ethiopian DHS (11,174 samples)	CatBoost, RF, XGBoost	97.08%	Identification of socioeconomic risk factors
Jain et al. [16]	Hemoglobinopathies	MCDM for formula selection	6388 (PGIMER), 939 (Kolkata)	TOPSIS, COPRAS, SECA	SCS BTT: 100% sensitivity (MCV < 80)	Inexpensive yet accurate tools for mass screening
Parishani & Rasti-Barzoki [17]	Classifier Selection	CWBCM with Confusion Matrix	6 datasets (COVID-19, diabetes, thyroid)	CWBCM, AHP, Entropy	CWBCM outperformed others	AHP and Entropy lack performance weighting nuance
Mustapha et al. [18]	Breast Cancer Screening	Supervised ML + MCDM	WBCD dataset (569 records)	AHP, TOPSIS, 10-fold CV	Evaluated holistically	Few models assess usability alongside accuracy
Stenwig et al. [19]	ICU Mortality Prediction	Explainable ML (SHAP)	eICU DB (200K+ patients)	RF, NB, LR, AdaBoost	Similar accuracy, different interpretability	Need for trustworthy and interpretable ML in healthcare

**Table 2:** Summary of Dataset Variables and Survey Questions.

#	Field Name	Question	Answer Format
1	Age	What is your age?	Enter your age in years
2	BMI	What is your Body Mass Index (BMI)?	Enter BMI value
3	Hemoglobin_Level (Hb)	What is your hemoglobin level?	Enter in g/dL
4	Family_History_Thalassemia	Do you have a family history of thalassemia?	Yes/No
5	Genetic_Marker_Presence	Is a genetic marker for thalassemia present?	Yes/No
6	Socioeconomic_Status	What is your socioeconomic status?	Low/Middle/High
7	Education_Level	What is your highest level of education completed?	No formal education/Primary/Secondary/Higher Secondary/Graduate
8	Residence	Do you live in an urban or rural area?	Urban/Rural
9	Parity	How many children have you given birth to?	Enter number
10	Carrier_Status	Are you a known carrier of thalassemia?	Yes/No
11	Hct	What is your hematocrit (Hct) level?	Enter in %
12	MCV	What is your Mean Corpuscular Volume (MCV)?	Enter in fL
13	MCH	What is your Mean Corpuscular Hemoglobin (MCH)?	Enter in pg
14	MCHC	What is your Mean Corpuscular Hemoglobin Concentration (MCHC)?	Enter in g/dL
15	RDW	What is your Red Cell Distribution Width (RDW)?	Enter in %
16	RBC count	What is your Red Blood Cell (RBC) count?	Enter in million cells/µL

**Table 3:** Patient Information and Blood Test Parameters.

ID	Age	BMI	Hb	Fam. Hist	Gen. Marker	SE Status	Education Level	Residence	Parity	Carrier	Hct	MCV	MCH	MCHC	RDW	RBC
1	36	24.0	11.8	No	1	Middle	Higher Secondary	Rural	0	Non-Car.	36.0	72.3	22.1	31.3	14.2	5.25
2	39	22.6	10.4	No	0	Low	Secondary	Urban	3	Non-Car.	36.8	69.9	23.7	33.3	12.3	5.34
3	22	21.9	9.3	Yes	1	Middle	Secondary	Rural	0	Non-Car.	30.8	62.7	24.6	31.0	11.6	4.99
4	18	19.6	12.2	No	0	Middle	Secondary	Urban	1	Non-Car.	29.0	63.7	22.3	33.6	14.0	4.55
5	27	22.5	9.1	No	0	Low	Secondary	Rural	1	Non-Car.	29.2	76.9	19.9	33.3	12.1	4.91
6	27	29.3	12.3	Yes	1	Middle	Secondary	Urban	3	Non-Car.	31.3	62.0	19.2	30.3	14.3	5.22
7	37	19.3	10.5	No	0	Low	Primary	Urban	3	Non-Car.	33.3	70.7	19.0	31.1	12.7	5.18
8	39	27.4	10.5	No	0	High	Primary	Rural	2	Carrier	32.8	62.9	20.3	34.0	11.2	5.87
9	31	22.0	11.5	No	0	Low	Primary	Urban	3	Non-Car.	32.4	67.4	23.0	31.0	12.5	4.95
10	36	22.6	10.5	No	0	Low	Primary	Urban	2	Carrier	29.2	75.5	23.1	31.7	12.1	4.96

**Table 4:** Saaty’s 1–9 Fundamental Scale of Relative Importance.

Intensity of Importance	Definition
1	Equal importance
2	Between equal and moderate importance
3	Moderate importance
4	Between moderate and strong importance
5	Strong importance
6	Between strong and very strong importance
7	Very strong importance
8	Between very strong and extreme importance
9	Extreme importance

**Table 5:** Missing Value Summary for All Features.

Feature	Missing	Feature	Missing
Patient_ID	0	Hct	0
Age	0	MCV	0
BMI	0	MCH	0
Hemoglobin_Level	0	MCHC	0
Family_History_Thalassemia	0	RDW	0
Genetic_Marker_Presence	0	RBC count	0
Socioeconomic_Status	0	Diagnosis	0
Education_Level	0	Residence	0
Parity	0	Carrier_Status	0

**Table 6:** Comparison of feature importance (Random Forest) and feature scores (SelectKBest).

Feature	Random Forest Importance	SelectKBest Score
Hct	0.103	4.309
RBC count	0.103	0.222
BMI	0.101	0.420
MCV	0.094	0.290
MCH	0.092	0.630
Hemoglobin Level	0.091	0.575
MCHC	0.089	1.429
RDW	0.088	1.336
Age	0.076	0.201
Education Level	0.038	2.256
Parity	0.037	0.090
Socioeconomic Status	0.024	1.064
Residence	0.017	0.129
Family History of Thalassemia	0.017	1.555
Genetic Marker Presence	0.016	0.045
Carrier Status	0.011	1.688

**Table 7:** Pairwise Comparison Matrix for AHP.

	Hct	MCHC	BMI	MCH	RDW	RBC Count	Hemoglobin	Family_Hist	Socioeco	MCV
Hct	1	3	5	4	3	5	7	6	4	5
MCHC	0.333	1	2	1	3	4	5	3	2	3
BMI	0.2	0.5	1	1	2	3	4	2	1	2
MCH	0.25	1	1	1	2	3	4	2	2	2
RDW	0.333	0.333	0.5	0.5	1	1	2	1	1	1
RBC count	0.2	0.25	0.333	0.333	1	1	3	2	2	2
Hemoglobin	0.143	0.2	0.25	0.25	0.5	0.333	1	4	3	3
Family_Hist	0.167	0.333	0.5	0.5	1	0.5	0.25	1	2	2
Socioeco	0.25	0.5	1	0.5	1	0.5	0.333	0.5	1	1
MCV	0.2	0.333	0.5	0.5	1	0.5	0.333	0.5	1	1

**Table 8:** Normalized AHP Pairwise Comparison Matrix.

	Hct	MCHC	BMI	MCH	RDW	RBC Count	Hemoglobin _Level	Family_ History_ Thalassemia	Socio- Economic _Status	MCV
Hct	0.3251	0.4027	0.4138	0.4174	0.1935	0.2655	0.2601	0.2727	0.2105	0.2273
MCHC	0.1084	0.1342	0.1655	0.1043	0.1935	0.2124	0.1858	0.1364	0.1053	0.1364
BMI	0.0650	0.0671	0.0828	0.1043	0.1290	0.1593	0.1486	0.0909	0.0526	0.0909
MCH	0.0813	0.1342	0.0828	0.1043	0.1290	0.1593	0.1486	0.0909	0.1053	0.0909
RDW	0.1084	0.0447	0.0414	0.0522	0.0645	0.0531	0.0743	0.0455	0.0526	0.0455
RBC count	0.0650	0.0336	0.0276	0.0348	0.0645	0.0531	0.1115	0.0909	0.1053	0.0909
Hemoglobin Level	0.0464	0.0268	0.0207	0.0261	0.0323	0.0177	0.0372	0.1818	0.1579	0.1364
Family History Thalassemia	0.0542	0.0447	0.0414	0.0522	0.0645	0.0265	0.0093	0.0455	0.1053	0.0909
Socioeconomic Status	0.0813	0.0671	0.0828	0.0522	0.0645	0.0265	0.0124	0.0227	0.0526	0.0455
MCV	0.0650	0.0447	0.0414	0.0522	0.0645	0.0265	0.0124	0.0227	0.0526	0.0455

**Table 9:** AHP-Derived Feature Weights.

Feature	AHP Weight
Hct	0.2989
MCHC	0.1482
MCH	0.1127
BMI	0.0991
Hemoglobin_Level	0.0683
RBC count	0.0677
RDW	0.0582
Family_History_Thalassemia	0.0534
Socioeconomic_Status	0.0508
MCV	0.0428

**Table 10:** Min-Max Normalized Values of Selected Features (First 10 Entries).

Entry	Hct	MCHC	MCH	BMI	Hemoglobin	RBC Count	RDW	Family Hist.	Socioeco. Status	MCV
1	0.8889	0.2167	0.5857	0.4783	0.8444	0.5000	0.9143	0.0	1.0	0.6150
2	0.9778	0.5500	0.8143	0.3565	0.5333	0.5600	0.3714	0.0	0.5	0.4950
3	0.3111	0.1667	0.9429	0.2957	0.2889	0.3267	0.1714	1.0	1.0	0.1350
4	0.1111	0.6000	0.6143	0.0957	0.9333	0.0333	0.8571	0.0	1.0	0.1850
5	0.1333	0.5500	0.2714	0.3478	0.2444	0.2733	0.3143	0.0	0.5	0.8450
6	0.2667	0.4333	0.3000	0.4130	0.2444	0.3333	0.0857	1.0	0.5	0.5350
7	0.1778	0.5833	0.2714	0.4696	0.2000	0.2867	0.6286	0.0	0.0	0.8650
8	0.9333	0.6500	0.7857	0.3869	0.5111	0.4067	0.6857	0.0	1.0	0.4250
9	0.5556	0.4000	0.5000	0.3826	0.5333	0.3667	0.7429	1.0	0.0	0.5050
10	0.2222	0.5167	0.6143	0.4565	0.6444	0.2267	0.7429	0.0	0.5	0.8850

**Table 11:** Relative Closeness and Final Risk Ranking (First 10 Entries).

ID	Relative Closeness	Risk Rank	Risk Category
1	0.7064	141	High Risk
2	0.7433	73	High Risk
3	0.3414	992	Medium Risk
4	0.2414	1162	Low Risk
5	0.2906	1094	Low Risk
6	0.2471	1157	Low Risk
7	0.3601	961	Medium Risk
8	0.6082	410	Medium Risk
9	0.4325	835	Medium Risk
10	0.2744	1115	Low Risk

**Table 12:** Performance Comparison of Machine Learning Models without AHP-TOPSIS.

Model	Accuracy (%)	Precision (%)	Recall (%)	F1-Score (%)	MCC (%)
Random Forest	86.44	85.79	87.34	86.56	72.89
XGBoost	**92.31**	**93.49**	**90.96**	**92.21**	**84.66**
CatBoost	88.97	88.69	89.33	89.01	77.94

**Table 13:** Performance Comparison of Machine Learning Models After AHP-TOPSIS (in %).

Model	Accuracy	Precision	Recall	F1-Score	MCC
Random Forest	94.58%	93.78%	95.48%	94.62%	89.16%
XGBoost	**99.28%**	99.10%	**99.46%**	**99.28%**	**98.55%**
CatBoost	98.73%	**99.45%**	98.01%	98.72%	97.48%

**Table 14:** Side-by-Side Performance Comparison of Machine Learning Models With and Without AHP-TOPSIS (in %).

Model	AHP-TOPSIS	Accuracy	Precision	Recall	F1-Score	MCC
Random Forest	Without	86.44	85.79	87.34	86.56	72.89
	With	**94.58**	**93.78**	**95.48**	**94.62**	**89.16**
XGBoost	Without	92.31	93.49	90.96	92.21	84.66
	With	**99.28**	**99.10**	**99.46**	**99.28**	**98.55**
CatBoost	Without	88.97	88.69	89.33	89.01	77.94
	With	**98.73**	**99.45**	**98.01**	**98.72**	**97.48**

**Table 15:** Quantitative comparison of the proposed AHP–TOPSIS–XGBoost framework with related MCDM–ML studies.

Study	Model/ Framework	Accuracy (%)	Precision (%)	Recall (%)	F1-Score (%)	ROC/AUC (%)
Rustam et al. [14]	CatBoost	97.08	97.09	97.05	97.06	99.9
Endalamaw et al. [13]	CatBoost + Ensemble (Anemia)	97.60	97.60	97.40	97.50	99.9
**Proposed Study**	**AHP + TOPSIS + XGBoost**	**99.28**	**99.10**	**99.46**	**99.28**	**99.96**

## Data Availability

The data underlying the findings of this study are available from the corresponding authors upon reasonable request and with proper justification.

## References

[1] Shafique F., Ali S., Almansouri T., Van Eeden F., Shafi N., Khalid M., Khawaja S., Andleeb S., ul Hassan M. (2021). Thalassemia, a human blood disorder. Braz. J. Biol..

[2] Singh P., Shaikh S., Parmar S., Gupta R. (2023). Current Status of *β*-Thalassemic Burden in India. Hemoglobin.

[3] Islam M., Kamruzzaman M., Sarker M., Riaaz R., Ilhan N. (2024). The Parental Perspective of Thalassemia in Bangladesh: Challenges for Prevention and Management of Thalassemia. Sch. J. App. Med. Sci..

[4] Ruangvutilert P., Phatihattakorn C., Yaiyiam C., Panchalee T. (2023). Pregnancy outcomes among women affected with thalassemia traits. Arch. Gynecol. Obstet..

[5] Hossain M.S., Mahbub Hasan M., Petrou M., Telfer P., Mosabbir A.A. (2021). The parental perspective of thalassaemia in Bangladesh: Lack of knowledge, regret, and barriers. Orphanet J. Rare Dis..

[6] Ghafor F., Ali T. (2024). Compulsory Pre-marital Thalassemia Screening to Mitigate the Burden of Thalassemia Major on Society and Healthcare System of Pakistan. Med. Sci. J. Adv. Res..

[7] Azhar N.A., Radzi N.A.M., wan Ahmad W.S.H.M. (2021). Multi-Criteria Decision Making: A Systematic Review. Rec. Adv. Electr. Electron. Eng. (Formerly Recent Patents Electr. Electron. Eng.).

[8] Karim R., Mahmud T., Hossain S., Hossain M.S., Zobaier A., Ahmed M., Sharmen N., Hossain M.S., Andersson K. (2023). A Belief Rule Based Decision Support System to Assess Multiple Disease Suspicion from Signs and Symptoms Under Uncertainty. Proceedings of the International Conference on Intelligent Computing & Optimization.

[9] Sharma D., Sridhar S., Claudio D. (2020). Comparison of AHP-TOPSIS and AHP-AHP methods in multi-criteria decision-making problems. Int. J. Ind. Syst. Eng..

[10] Tiwari R. (2023). Explainable AI (xai) and its applications in building trust and understanding in ai decision making. Int. J. Sci. Res. Eng. Manag..

[11] Dey P., Mahmud T., Hossain M.S., Andersson K. (2023). Improving Pneumonia Detection with Deep Learning Models: Insights from Chest X-Rays. Proceedings of the International Conference on Intelligent Computing & Optimization.

[12] Chowdhury T., Mahmud T., Mallik A., Mujdhalifa N.A., Barua K., Sharmen N., Hossain M.S., Andersson K. (2023). Development of an Android-Based Pneumonia Detection App: Bridging Healthcare Gaps. Proceedings of the International Conference on Intelligent Computing & Optimization.

[13] Endalamaw B., Abuhay T.M., Shibabaw D. Predicting the Level of Anemia among Ethiopian Pregnant Women using Homogeneous Ensemble Machine Learning Algorithm. Proceedings of the 2nd Deep Learning Indaba-X Ethiopia Conference 2021.

[14] Rustam F., Ashraf I., Jabbar S., Tutusaus K., Mazas C., Barrera A.E.P., de la Torre Diez I. (2022). Prediction of *β*-thalassemia carriers using complete blood count features. Sci. Rep..

[15] Göl M., Aktürk C., Talan T., Vural M.S., Türkbeyler İ.H. (2025). Predicting malnutrition-based anemia in geriatric patients using machine learning methods. J. Eval. Clin. Pract..

[16] Jain A.K., Sharma P., Saleh S., Dolai T.K., Saha S.C., Bagga R., Khadwal A.R., Trehan A., Nielsen I., Kaviraj A. (2024). Multi-criteria decision making to validate performance of RBC-based formulae to screen *β*-thalassemia trait in heterogeneous haemoglobinopathies. BMC Med. Inform. Decis. Mak..

[17] Parishani M., Rasti-Barzoki M. (2024). CWBCM method to determine the importance of classification performance evaluation criteria in machine learning: Case studies of COVID-19, Diabetes, and Thyroid Disease. Omega.

[18] Mustapha M.T., Ozsahin D.U., Ozsahin I., Uzun B. (2022). Breast cancer screening based on supervised learning and multi-criteria decision-making. Diagnostics.

[19] Stenwig E., Salvi G., Rossi P.S., Skjærvold N.K. (2022). Comparative analysis of explainable machine learning prediction models for hospital mortality. BMC Med. Res. Methodol..

[20] Akhiat Y., Manzali Y., Chahhou M., Zinedine A. (2021). A new noisy random forest based method for feature selection. Cybern. Inf. Technol..

[21] Tislenko M., Gaidel A., Kupriyanov A. Comparison of feature selection algorithms for Data classification problems. Proceedings of the 2022 VIII International Conference on Information Technology and Nanotechnology (ITNT).

[22] Gyani J., Ahmed A., Haq M.A. (2022). MCDM and various prioritization methods in AHP for CSS: A comprehensive review. IEEE Access.

[23] Yu D., Kou G., Xu Z., Shi S. (2021). Analysis of Collaboration Evolution in AHP Research: 1982–2018. Int. J. Inf. Technol. Decis. Mak..

[24] Handayani A., Farikhin F., Surarso B. (2023). Statisticam approaches for consistency index in analytical hierarchy process. Aksioma: J. Mat. Pendidik. Mat..

[25] Talekar B. (2020). A Detailed Review on Decision Tree and Random Forest. BIoscience Biotechnol. Res. Commun..

[26] Ali Z.A., Abduljabbar Z.H., Tahir H.A., Sallow A.B., Almufti S.M. (2023). eXtreme gradient boosting algorithm with machine learning: A review. Acad. J. Nawroz Univ..

[27] Kulkarni C.S. (2022). Advancing Gradient Boosting: A Comprehensive Evaluation of the CatBoost Algorithm for Predictive Modeling. J. Artif. Intell. Mach. Learn. Data Sci..

[28] Abyad A. (2020). The pareto principle: Applying the 80/20 rule to your business. Middle East J. Bus..

[29] Çorbacıoğlu Ş.K., Aksel G. (2023). Receiver operating characteristic curve analysis in diagnostic accuracy studies: A guide to interpreting the area under the curve value. Turk. J. Emerg. Med..

[30] Pinho M., Moura A. (2021). A decision support system to solve the problem of health care priority-setting. J. Sci. Technol. Policy Manag..

